# Novel Aminoguanidine Hydrazone Analogues: From Potential Antimicrobial Agents to Potent Cholinesterase Inhibitors

**DOI:** 10.3390/ph14121229

**Published:** 2021-11-26

**Authors:** Martin Krátký, Šárka Štěpánková, Klára Konečná, Katarína Svrčková, Jana Maixnerová, Markéta Švarcová, Ondřej Janďourek, František Trejtnar, Jarmila Vinšová

**Affiliations:** 1 Department of Organic and Bioorganic Chemistry, Faculty of Pharmacy in Hradec Králové, Charles University, Akademika Heyrovského 1203, 50005 Hradec Králové, Czech Republic; komloova@seznam.cz (M.Š.); vinsova@faf.cuni.cz (J.V.); 2 Department of Biological and Biochemical Sciences, Faculty of Chemical Technology, University of Pardubice, Studentská 573, 53210 Pardubice, Czech Republic; sarka.stepankova@upce.cz (Š.Š.); katarina.svrckova@upce.cz (K.S.); 3 Department of Biological and Medical Sciences, Faculty of Pharmacy in Hradec Králové, Charles University, Akademika Heyrovského 1203, 50005 Hradec Králové, Czech Republic; konecna@faf.cuni.cz (K.K.); jando6aa@faf.cuni.cz (O.J.); 4 Department of Pharmacology and Toxicology, Faculty of Pharmacy in Hradec Králové, Charles University, Akademika Heyrovského 1203, 50005 Hradec Králové, Czech Republic; maixj6a1@faf.cuni.cz (J.M.); trejtnarf@faf.cuni.cz (F.T.); 5 Department of Chemistry, Faculty of Science, J. E. Purkinje University, Pasteurova 3632/15, 40096 Ústí nad Labem, Czech Republic

**Keywords:** acetylcholinesterase, aminoguanidine, antimicrobial activity, butyrylcholinesterase, cytotoxicity, enzyme inhibition, hydrazones, molecular docking, salicylaldehydes

## Abstract

A series of thirty-one hydrazones of aminoguanidine, nitroaminoguanidine, 1,3-diaminoguanidine, and (thio)semicarbazide were prepared from various aldehydes, mainly chlorobenzaldehydes, halogenated salicylaldehydes, 5-nitrofurfural, and isatin (yields of 50–99%). They were characterized by spectral methods. Primarily, they were designed and evaluated as potential broad-spectrum antimicrobial agents. The compounds were effective against Gram-positive bacteria including methicillin-resistant *Staphylococcus aureus* with minimum inhibitory concentrations (MIC) from 7.8 µM, as well as Gram-negative strains with higher MIC. Antifungal evaluation against yeasts and *Trichophyton mentagrophytes* found MIC from 62.5 µM. We also evaluated inhibition of acetylcholinesterase (AChE) and butyrylcholinesterase (BuChE). The compounds inhibited both enzymes with IC_50_ values of 17.95–54.93 µM for AChE and ≥1.69 µM for BuChE. Based on the substitution, it is possible to modify selectivity for a particular cholinesterase as we obtained selective inhibitors of either AChE or BuChE, as well as balanced inhibitors. The compounds act via mixed-type inhibition. Their interactions with enzymes were studied by molecular docking. Cytotoxicity was assessed in HepG2 cells. The hydrazones differ in their toxicity (IC_50_ from 5.27 to >500 µM). Some of the derivatives represent promising hits for further development. Based on the substitution pattern, it is possible to modulate bioactivity to the desired one.

## 1. Introduction

Molecular hybridization approach belongs to the most frequent and perspective tools in medicinal chemistry and drug design. Combination of various bioactive scaffolds in one molecular entity offers many advantages [1].

Aminoguanidine (AG; hydrazinecarboximidamide) inhibits selectively inducible nitric oxide synthase and scavenges reactive oxygen species, thus being antioxidant agent protecting various cells and tissues from oxidative stress. Moreover, AG was the first inhibitor of the advanced glycation pathway that plays a crucial role in the pathogenesis of late diabetes mellitus complications (angiopathy, neuropathy, nephropathy, etc.). It is able to scavenge carbonyl reactive intermediates [2,3]. In addition, AG and its derivatives also showed multiple anti-inflammatory properties [4,5,6]. However, the therapeutical potential is abolished due to nucleophilic reaction with pyridoxal phosphate (active form of vitamin B6) leading to its depletion and in vivo deficiency. It has been suggested that the formation of AG hydrazones does not influence levels of this vital molecule [3].

The condensation of AG with both “simple” aldehydes/ketones and carbonyl compounds with an intrinsic activity has provided conjugates with a significant antibacterial and antifungal activity covering a broad spectrum of Gram-positive, Gram-negative and fungal species such as drug-resistant strains including methicillin-resistant Staphylococcus aureus (MRSA) [5,6,7,8,9]. Replacement of AG by 1,3-diaminoguanidine is tolerated and vice versa [9]. Apart from direct killing of bacteria, hydrazones of AG are capable to inhibit efflux pumps, thus restoring activity of antibiotics and helping overcome acquired resistance, as demonstrated for fluoroquinolone norfloxacin against *Staphylococcus aureus* [10].

Moreover, aromatic hydrazones obtained from AG have been also proposed as antiparasitic [11], antitubercular [12], and antiplatelet [13] agents. They are also active against phytopathogenic fungi [14], and, importantly, they exhibited cytotoxic, antiproliferative, and pro-apoptotic, but not genotoxic properties [3,5,7,8,15].

Salicylaldehyde-based hydrazones and imines represent a class of compounds with a great variety of biological activities. For design of this study, antibacterial, antifungal, and cytotoxic actions are of a special interest [16,17,18,19].

Neurotransmitter acetylcholine (ACh) is mainly synthetized from acetyl coenzyme A and choline by choline acetyltransferase. ACh modulates many vital functions and its effects are exerted through muscarinic and nicotinic cholinergic receptors. Action of ACh on synapses is terminated via hydrolytic cleavage catalyzed by either of two structurally similar enzymes–acetylcholinesterase (AChE) and butyrylcholinesterase (BuChE). Drugs boosting ACh neurotransmission have been reported to have a beneficial effect on some types of dementia including Alzheimer’s disease, glaucoma, myasthenia gravis, atonia, and tachycardia. They have been used in prophylaxis of nerve paralytic agents. Among various mechanisms of action, AChE and BuChE inhibitors are of a special interest [20].

To our best knowledge, no systematic study evaluating antimicrobial activity of hydrazones obtained from halogenated salicylaldehydes and AG or its isosteres and derivatives has been published. As a part of our ongoing research, we designed, prepared, and screened them as potential antimicrobial agents primarily; then, they were repurposed as inhibitors of acetylcholinesterase (AChE) and butyrylcholinesterase (BuChE) and we evaluated toxicity for eukaryotic cell line. In addition, we also investigated hydrazones of 5-nitro five-membered ring heterocycles-2-carbaldehydes systematically in a similar way as a result of a structure-based design strategy.

## 2. Results and Discussion

### 2.1. Design and Synthesis

**Figure 1:**
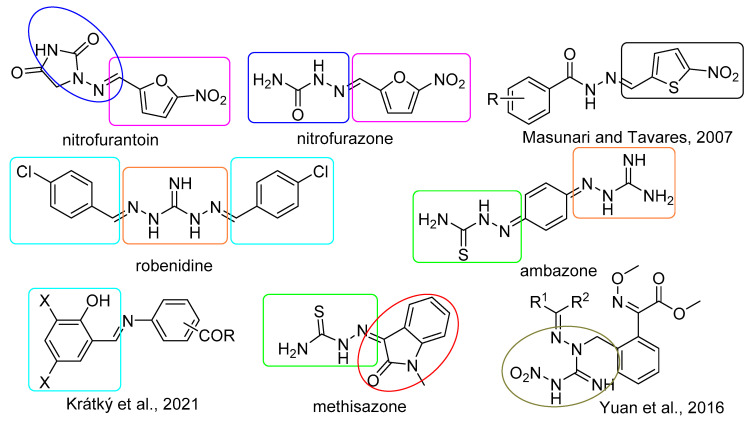
Structural motifs of known bioactive compounds used for design of targeted derivatives (aminoguanidine and 1,3-diaminoguanidine; nitroaminoguanidine; semicarbazone; thiosemicarbazone; 5-nitrofurylidene; (5-nitrothiophen-2-yl)methylidene; halogenobenzylidene; isatin).

The scaffolds used for design via molecular hybridization approach is depicted in Figure 1. In addition to bioactive AG and 1,3-diaminoguanidine derivatives summarized briefly in Introduction section, we utilize knowledge about antimicrobial nitrofuran drugs nitrofurantoin and nitrofurazone (antibacterial, antifungal, antiprotozoal agents), anti-MRSA 5-nitrothiophene analogues of antimicrobial drug nifuroxazide [21], antibiotic robenidine used mainly in control of *Coccidia* infections in poultry and rabbit suppression also drug-resistant Gram-positive cocci [22], topical antibacterial drug ambazone with anticancer properties [23], antiviral agent methisazone [24], as well as our recent 3,5-dihalogenosalicylidene aminobenzoic acid derivatives identified as potent compounds against Gram-positive bacteria, yeasts and molds [17,18]. Nitroaminoguanidines are effective as inhibitors of phytopathogenic fungi [25] and mycobacteria [26].

Initially, inspired by robenidine and its highly antibacterial active analogues [9,22], we prepared 4-chlorobenzylideneaminoguanidine **1d**, its positional isomers **1b** and **1c** and unsubstituted benzylidene analogue **1a** for comparison. Then, based on our positive experience with salicylic derivatives [16,17,18,19], we designed and synthesized analogues with introduced 2-hydroxy group into chlorobenzaldehydes (**1e**–**1h**) to investigate various positional isomers. Analogously to our previous work, we involved also 3,5-dihalogenosalicylaldehydes, particularly iodinated ones (**1i**–**1m**). Keeping in mind a known diverse bioactivity of isatin, 5-nitrofurfural and 5-nitrothiophen-2-carbaldehyde-derived imines and hydrazones, we also prepared their hydrazones with AG (**1p**, **1n**, and **1o**, respectively).

**Figure 2:**
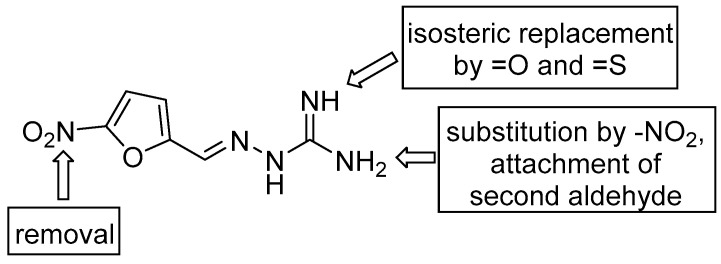
Design of 5-nitrofuran-based aminoguanidine analogues of **1n**.

**Figure 3:**
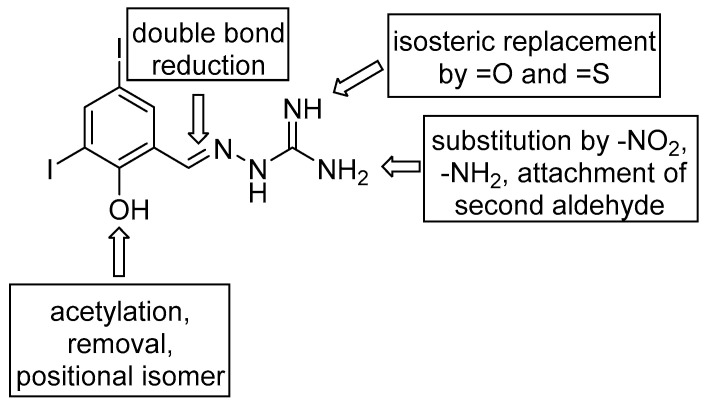
Design of 3,5-diiodosalicylidene-AG **1m** analogues.

After biological assessment of this initial series, reflecting structure-activity relationships that were found, we prepared additional derivatives. 5-Nitrofurylidene analogues with modified “amino part” were synthesized with respect to the antibacterial and almost selective AChE inhibition produced by **1n** (Figure 2), while me-too approach of **1m** initiated by its inhibitory properties for both AChE and BuChE covers both aldehyde (*O*-acetylation: **2a**, 4-OH isomer: **2b**, OH group removal: **2c**, double bond reduction: **2d**) and aminoguanidine substructures (Figure 3). Modification of AG consists in introduction of NO_2_ group (nitroaminoguanidines), second NH_2_ group enabling attachment of an additional aldehyde, and formally isosteric replacement of =NH group by =O (semicarbazones) and =S (thiosemicarbazones).

**Figure 4:**
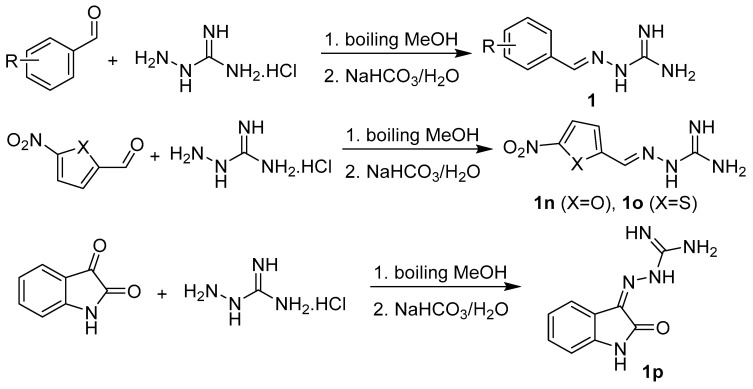
Synthesis of initial series of benzaldehyde/salicylaldehyde/isatin/5-nitroheteroarene-2-carbaldehyde-based aminoguanidines **1** (R = H (**1a**), 2-/3-/4-Cl (**1b**, **1c**, and **1d**, respectively), 2-OH-3-Cl (**1e**), 2-OH-4-Cl (**1f**), 2-OH-5-Cl (**1g**), 2-OH-6-Cl (**1h**), 2-OH-5-I (**1i**), 3,5-Cl_2_ (**1j**), 5-Cl-3-Br (**1k**), 5-Cl-3-I (**1l**), 3,5-I_2_ (**1m**)).

**Figure 5:**
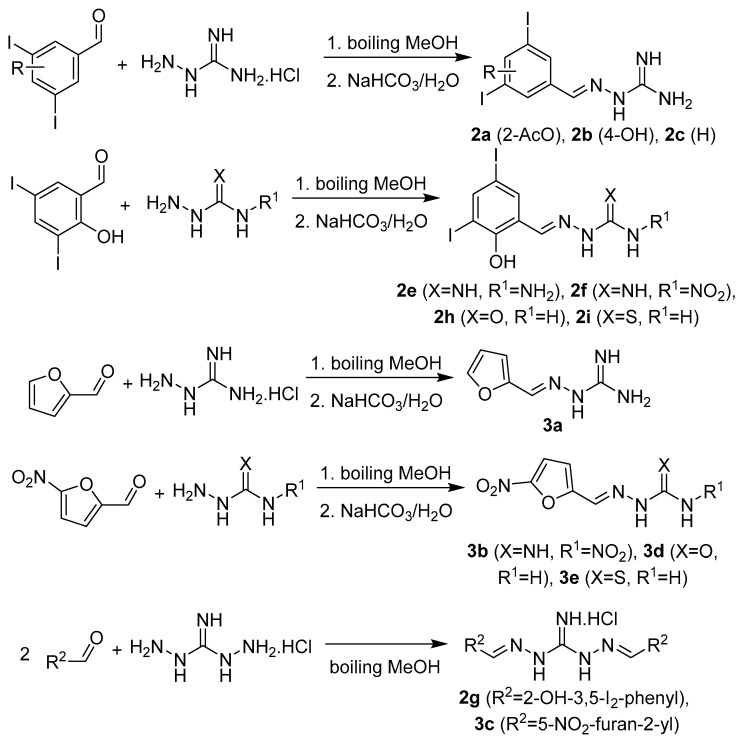
Synthesis of additional series.

Hydrazones were prepared by treatment of amino compound (aminoguanidine hydrochloride, nitroaminoguanidine, 1,3-diaminoguanidine hydrochloride, thiosemicarbazide, or semicarbazide hydrochloride; 1 mmol) with a mild excess of appropriate aldehyde or ketone (1.1 of equivalents or 2.2 of equivalents for disubstituted 1,3-diaminoguanidines **2g** and **3c**) in methanol under reflux for 3 h (Figure 4 and Figure 5). In some cases, the crystallization was initiated by addition of 5% sodium bicarbonate. Due to high solubility of **3a**, its synthesis was realized in water under reflux for 6 h and the solution was basified using solid sodium carbonate. The yields ranged from 65% to 99%. Lower yields were obtained for benzylidene derivative **1a** (78%) due to higher water solubility, lipophilic ester **2a** (74%), 3,5-diiodobenzylidene analogue **2c** (80%), and the only sole ketone (isatin) derivative **1p** (65%). The derivatives of 1,3-diaminoguanidine were isolated as hydrochlorides without basification (**2e**, **2g**, and **3c**) with yields of 50–93%. For the reaction providing monosubstituted 1,3-diaminoderivative **2e**, only 0.95 of equivalents of the aldehyde was used.

**Figure 6:**
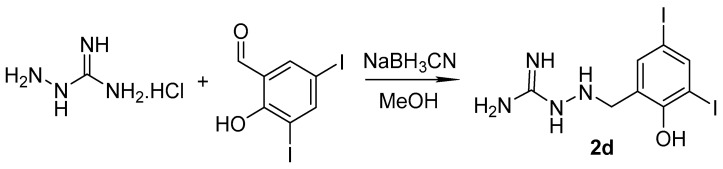
Reductive amination.

The analogue **2d** without double bond was prepared from aminoguanidine hydrochloride (1 mmol) dissolved in MeOH that was mixed successively with 3,5-diiodosalicylaldehyde (1.1 mmol) and sodium cyanoborohydride (1.6 mmol) (Figure 6). After 48 h at rt, the reaction mixture was diluted with 5% sodium bicarbonate and let to stir over night. The product was isolated with yield of 82%.

**Figure 7:**
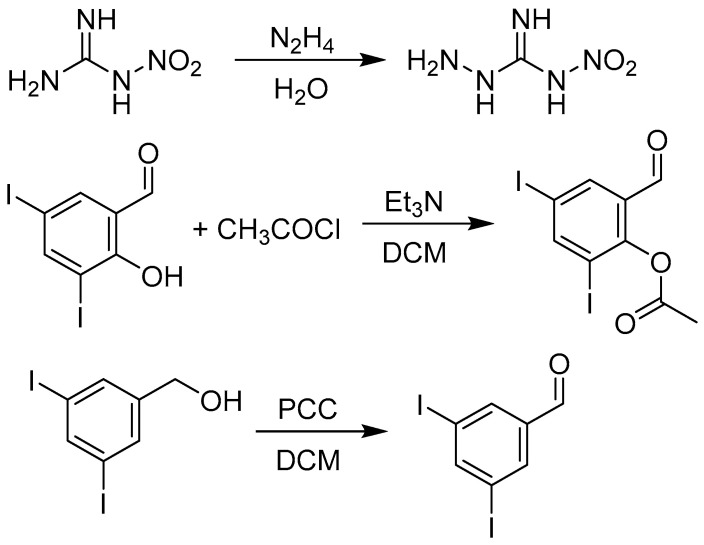
Preparation of synthetic precursors (PCC: pyridinium chlorochromate; DCM: dichloromethane).

Some precursors were prepared in-house (Figure 7). Nitroaminoguanidine was prepared from nitroguanidine and a slight excess of hydrazine hydrate (1.2 of eq.) with 72% yield. 2-Formyl-4,6-diiodophenyl acetate was synthesized from 3,5-diiodosalicylaldehyde and acetyl chloride in the presence of triethylamine (yield 93%). The treatment of the aldehyde with acetic anhydride with a catalytic amount of sulfuric acid under reflux gave “triacetylated” 2-acetoxy-3,5-diiodophenyl)methylene diacetate as the main product. 3,5-Diiodobenzaldehyde was prepared almost with quantitative yield from 3,5-diiodophenylmethanol using pyridinium chlorochromate.

The products were characterized by ^1^H and ^13^C NMR spectroscopy, IR spectra and melting points, the purity was checked additionally by elemental analysis. From total thirty-one compounds, fifteen were original (**1e**, **1h**, **1i**, **1k**–**1o**, **2a**–**2g**). NMR spectra of the representative compounds are illustrated in supplementary material (Figures S1–S10).

### 2.2. Microbiology

#### 2.2.1. Antibacterial Activity

Based on the literature, we evaluated antibacterial activity of **1**–**3** initially, using the microdilution broth method according to the EUCAST guidelines against four Gram-positive strains: *Staphylococcus aureus* ATCC 29213, methicillin-resistant *Staphylococcus aureus* (MRSA) ATCC 43300, *Staphylococcus epidermidis* ATCC 12228, *Enterococcus faecalis* ATCC 29212; and four Gram-negative strains: *Escherichia coli* ATCC 25922, *Klebsiella pneumoniae* ATCC 10031, *Acinetobacter baumannii* ATCC 19606, and *Pseudomonas aeruginosa* ATCC 27853. Results are presented in Table 1. Piperacillin and parent amino compounds (including AG, 1,3-diaminoguanidine, nitroaminoguanidine, semicarbazide, and thiosemicarbazide) were involved for activity comparison. Two compounds (**1c**, **1d**) were not evaluated due to solubility problems.

The parent amino compounds showed all MIC values above 500 µM (not involved in Table 1), so the activity strongly depends on aldehyde/ketone-derived part of the molecules. Ten target compounds avoided antibacterial properties, i.e., their MIC values were ≥500 µM (**1d**, **1e**, **1m**, **1p**, **2d**, **2f**, **2g**, **3a**–**3c**; not included in Table 1). Beta-lactam drug piperacillin showed higher activity against MSSA, *E. faecalis*, *E. coli* as well as *P. aeruginosa*, but not against *S. epidermidis*, *K. pneumoniae* and, of course, MRSA.

Regarding compounds **1**–**3**, Gram-positives share a higher susceptibility with MIC from 7.8 µM, while Gram-negative pathogens were significantly more resistant with MIC of ≥62.5 µM. The genus Staphylococcus was the most sensitive, importantly, without significant differences in susceptibility between MSSA and MRSA strains. Of Gram-negative species, *P. aeruginosa* and *A. baumannii* were more resistant (MIC from 125 µM).

The highest activity was associated with the presence of (5-nitrothiophen-2-yl)methylidene (especially against Gram-negative species; **1o**), 5-nitrofurylidene (**1n**, nitrofurazone **3d**, and **3e**) and 3,5-diiodobenzylidene (**2c**) moieties conferring all a broad-spectrum activity. Focusing on amine part, the derivatives of nitroaminoguanidine and disubstituted 1,3-diaminoguanidines avoided antibacterial activity.

Among benzylidene derivatives, the introduction of any additional substituent into the 3,5-diiodinated molecule **2c** resulted in completely abolished (2-OH: **1m**) or decreased activity (4-OH: **2b**, 2-AcO: **2a**). Interestingly, 3,5-diiodosalicylidene in combination with 1,3-diaminoguanidine is superior against staphylococci (**2c**; MIC of 31.25–125 µM). For antibacterial action, 5- or 3,5-disubstitution of salicylaldehyde are optimal with 5-chlorosalicylidene superiority (MIC from 62.5 µM). In contrast to our previous findings [16,17,18], neither heavier halogens nor 3,5-dihalogenation is not associated with an improved potency.

#### 2.2.2. Antifungal Activity

Parent amino compounds together with their derivatives **1**–**3** and triazole drug fluconazole were evaluated against eight human pathogenic fungi: *Candida albicans* ATCC 24443, *Candida krusei* ATCC 6258, *Candida parapsilosis* ATCC 22,019 and *Candida tropicalis* ATCC 750. *Aspergillus fumigatus* ATCC 204305, *Aspergillus flavus* CCM 8363, *Lichtheimia corymbifera* CCM 8077, and *Trichophyton interdigitale* ATCC 9533 (Table 2). 

The parent compounds lack any antifungal action (MIC >500 µM) as well as many of the investigated derivatives with MIC of ≥500 µM (**1a**, **1e**–**1h**, **1j**, **1m**, **1n**, **1p**, **2b**, **2c**, **2f**, **2h**, **2i**, **3a**–**3f**; not reported in Table 2). Two compounds were not evaluated due to solubility issues (chlorobenzylidene derivatives **1b** and **1c**).

When focused on fungal species, *L. corymbifera* and both strains of *Aspergillus* showed a complete resistance to the investigated molecules (data not shown). Contrarily, *T. interdigitale* as the most susceptible strain was supressed by fifteen compounds from the concentration of 62.5 µM with **1i**, **1l**, **2a**, **2d**, and **2e** superiority. Their MIC values are comparable to fluconazole; however, for fluconazole it means 50% inhibition of growth, while for our compounds a complete inhibition. Thus, even with similar numeral values, the derivatives are more potent.

*Candida* species showed a uniform susceptibility with MIC values of 125–500 µM. These strains were inhibited predominantly by four iodinated (**1i**, **1l**, **2a**, and **2e**) compounds and 3-bromo-5-chlorosalicylidene analogue **1k**.

*N*’-(2-Hydroxy-3,5-diiodobenzylidene)hydrazinecarboximidhydrazide **2e** was identified as the most potent and broad-spectrum antimycotic agents. However, the antifungal potency of **1**–**3** generally remained below expectation [7].

### 2.3. Inhibition of Acetyl- and Butyrylcholinesterase

After outcomes of antimicrobial activity, this follow-up screening was inspired by previous sporadic publication reporting compounds based on AG scaffold as potential AChE [27,28,29] and BuChE [29,30] inhibitors. Additionally, hydrazones [31,32] as well as salicylic compounds [31,33] have shown inhibition of these enzymes. The derivatives **1**–**3** together with their parent compounds were tested for their ability to suppress the function of AChE from electric eel (EeAChE) and BuChE from equine serum (EqBuChE) using Ellman’s method (Table 3). 

Inhibitor potency is expressed as the concentration resulting in 50% inhibition of enzymatic activity (IC_50_). Selectivity indexes (SI) were calculated to quantify the preferential affinity for AChE or BuChE. SI is defined as the ratio of IC_50_ for AChE/IC_50_ for BuChE (Table 3). Values above 1 indicate stronger inhibition of BuChE and vice versa. Clinically used cholinesterases (ChEs) inhibiting drugs galantamine and rivastigmine were included as standards. 

Most of the derivatives **1**–**3** exhibited a dual inhibition of both ChEs, nitro group containing derivatives **2f** and **3b**–**3e** being an exception with IC_50_ for BuChE over 500 µM. AChE was inhibited more consistently with IC_50_ values of 17.95–54.93 µM with isatin derivative **1p** superiority. Substitution of benzylidene derivative **1a** by chlorine alone or in a combination with 2-OH group did not improve activity generally (**1b**–**1h**, **1j**). On the other hand, 5-iodosalicylidene **1i** and 3,5-dihalogenosalicylidene scaffold, where at least one halogen is bromine or iodine, resulted in improved potency (**1m**, and especially **1k** and **1l**). The *O*-acetylation, OH group removal, insertion of NH_2_ or NO_2_ group into aminoguanidine part of the molecule as well as its replacement by semicarbazide are tolerated (**1m** vs. **2a**, **2c**, **2e**, **2f**, and **2h**). Contrarily, double bond reduction (**2d**), OH group positional isomer **2b**, introduction of second 3,5-diiodosalicylideneamino group (**2g** with the highest IC_50_ of 54.93 µM) or AG replacement by thiosemicarbazide (**2i**) are detrimental. Among nitro heterocycle-based hydrazones, the best activity is associated with the presence of 5-nitrofurylidene moiety on (nitro)aminoguanidine (**1n** and **3b**); the replacement by thiophene is acceptable (**1n** vs. **1o**), while removal of NO_2_ as well as duplication of 5-nitrofurylideneamino moiety decreased activity (**1n** vs. **3a** and **3c**). The substitution of benzylidene derivatives **1a** resulted in more potent inhibitors in the case of iodine (**1i**, **1l**, **1m**, **2a**, and **2c**) and bromine (**1k**), mainly together with 2-phenolic group. Interestingly, the isomeric chlorobenzaldehyde conjugates **1b**–**1d** exhibited similar inhibition. 

BuChE was inhibited in a broad range of concentrations with IC_50_ from 1.69 µM. Based on IC_50_ values, we can classify compounds into three groups: (1) highly active inhibitors with IC_50_ lower than 20 µM (iodosalicylidene derivatives **2e**, **1m**, **2a**, **1l**, **1i**, and **2g**, other halogenated salicylidene analogues: 3-Cl **1e**, 3-Br-5-Cl **1k**, 4-chlorobenzylidene **1d**, and isatine-derived hydrazone **1p**); (2) compounds with moderate activity (**1a**–**1c**, **1f**–**1h**, **1j**, **2b**–**2d**, **2h**, **2i**, and **3a**); and (3) derivatives with low (**1n**, **1o**; 204–286 µM) or virtually no activity (**2f**, **3b**–**3e**, >500 µM). In general, presence of NO_2_ group either in arylidene or amino part led to substantial drop of BuChE inhibition (as illustrated by pairs **1n** vs. **3a** and **1n** vs. **2b**). Focusing on modification of 3,5-diiodosalicylidene derivative **1m** (4.52 µM), the most potent agent from the initial series, the replacement of 5-iodine by chlorine (**1l**), *O*-acetylation (**2a**), introduction of additional 3,5-diiodosalicylideneamino (**2g**) or NH_2_ group (**2e**) are tolerated, although only the last modification provided improved activity (2.1×, IC_50_ of 1.69 µM, i.e., the most potent compound against BuChE in this study). On the other hand, OH group removal (**2c**), change of position (**2b**), hydrazone bond reduction (**2d**), replacement of AG by nitroaminoguanidine (**2f**) or (thio)semicarbazide (**2h**, **2i**) are disadvantageous. Among salicylidene derivatives, inhibitory potency is modulated positively by a presence of two halogens of which at least one is not a chlorine; an increasing molecular mass of halogen(s) contributes to lower IC_50_ (**1g** vs. **1i**, **1j** vs. **1k** vs. **1l** vs. **1m**; **1i** vs. **1m**). The substitution of **1a** has an ambiguous effect on activity: several compounds were more potent (**1d**–**1f**, **1i**, **1k**–**1m**, and **2a**), several comparable (**1c**, **1g**, **2b**), remaining ones were unfavorable. Interestingly, 3,5-diiodo substitution itself is not beneficial since salicylic hydroxyl is essential for high BuChE inhibition (**1m** vs. **2c**). The introduction of OH group into isomeric chlorobenzylidene analogues, where 4-chloro one **1d** was the most potent, has no clear effect; this change is advantageous for 2-Cl one (**1b** vs. **1h**) and one of 3-Cl-benzylidine modification (**1c** vs. **1e**, 7.4 ×), but not for 4-Cl (**1d** vs. **1f**). Finally, isosteric replacement of oxygen by sulfur brought a drop of activity (**1n** vs. **1o**, **2h** vs. **2i**).

The selectivity for each particular ChEs can be described on the basis of selectivity indexes. Seven compounds inhibited BuChE significantly more effectively (SI >3: **1d**, **1e**, **1l**, **1m**, **2a**, **2g**, and above all **2e** with SI = 16.7). On the other hand, five hydrazones are highly selective AChE inhibitors (SI ≤ 0.1: **2f**, and **3b**–**3e**), closely followed by remaining nitro compounds (**1n**, **1o**). Thus, presence of NO_2_ group in either arylidene or aminoguanidine portion results in selective AChE inhibition, which is demonstrated illustratively by pair **2e** and **2f**, where replacement of NO_2_ with NH_2_ led to more than 239.5-fold increased selectivity for BuChE. This modification substantially alters both electronic properties and lipophilicity. Other clear structural features modulating SI are presence of 2-chlorobenzylidene (**1b**, **1h**) leading to higher selectivity for AChE compared to its isomers. Focusing on 3,5-halogenosalicylidenes, heavier halogens (**1j**–**1m**) turn selectivity to BuChE. Modification of 3,5-diiodosalicylidene compound **1m** provided mainly compounds with reduced BuChE selectivity (phenolic group removal, acetylation, hydrazone reduction, positional isomer, analogous nitroaminoguanidine, semicarbazone and thiosemicarbazone: **2c**, **2a**, **2d**, **2b**, **2f**, **2h**, and **2i**, respectively), only the homologue with additional amine group **2e** was more selective. Thus, based on the substitution, it is possible to modulate selectivity for both ChEs.

Drawing a comparison to the azepine drug galantamine, our derivatives are one order of magnitude less active against AChE, but the most active hydrazones produced comparable IC_50_ values for BuChE (**1e**, **1l**, **1m**, **2a**, and especially **2e**). Remarkably, the dual ChEs inhibitor rivastigmine showed higher or comparable IC_50_ against AChE than hydrazones **1**–**3**, whereas there was no dominant trend for BuChE. We prepared a number of more active derivatives (**1d**–**1f**, **1i**, **1k**–**1m**, **1p**, **2a**, **2e**, **2g**, **2h**), the remaining ones were slightly or clearly (**1b**, **1n**, **1o**, **2c**, **2f**, **3a**–**3e**) less active.

Notably, all precursor amino compounds significantly and selectively inhibited AChE with IC_50_ values of 41.71–75.14 µM; AG was least effective. BuChE was inhibited to a lesser extent (from 283.32 µM for 1,3-diaminoguanidine, the most potent precursor ChEs inhibitor), while introduction of nitro group resulted in a complete loss of activity (IC_50_ >500 µM). Importantly, their modification to form hydrazones led to substantially better activity against BuChE, by up to two orders of magnitude; nitroaminoguanidines **2f** and **3b** were exceptions reflecting the potency of this scaffold along with some 5-nitrofurylidene analogues (**3c**–**3e**). Conversion of AG, nitroaminoguanidine and semicarbazide to their hydrazones is also advantageous for uniformly improved AChE inhibition (up to 4.2-fold for **1p**), whereas results for thiosemicarbazide and 1,3-diaminoguanidine are ambiguous.

These results qualify the presented compounds to be considered as potential agents for treatment of neurodegenerative disorders, myasthenia gravis, and other diseases where boosting of cholinergic neurotransmission is an option.

#### 2.3.1. Type of Inhibition

Based on promising inhibition data, we evaluated type of inhibition of the most active hydrazones. In general, the reversible enzyme inhibitors can be classified as competitive, non-competitive, uncompetitive, or mixed type. The type of inhibition could be distinguished using the Lineweaver–Burk plot [34] and corresponding comparison of two kinetic parameters: maximum velocity (V_m_) and Michaelis constant (K_M_) of inhibited and uninhibited reactions. Based on changes of these parameters and intercept of lines in Lineweaver-Burk plot, type of inhibition is revealed. 

**Figure 8:**
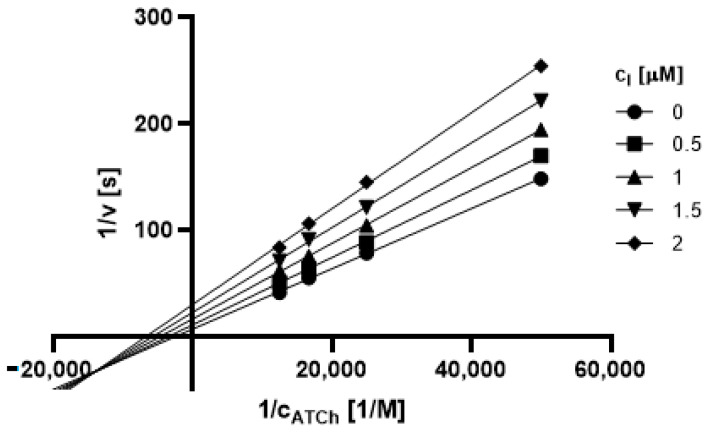
Lineweaver–Burk plot for **1n** and AChE inhibition (ATCh = acetylthiocholine).

**Figure 9:**
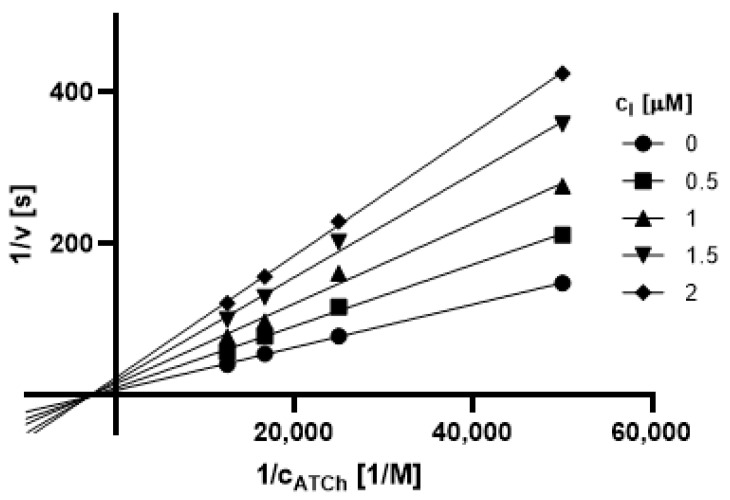
Lineweaver–Burk plot for **1p** and AChE inhibition (ATCh = acetylthiocholine).

**Figure 10:**
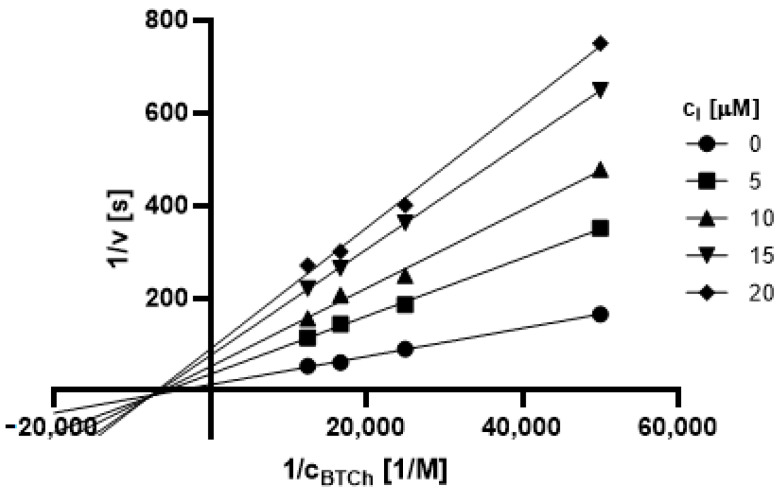
Lineweaver–Burk plot for **1p** and BuChE inhibition (BTCh = butyrylthiocholine).

**Figure 11:**
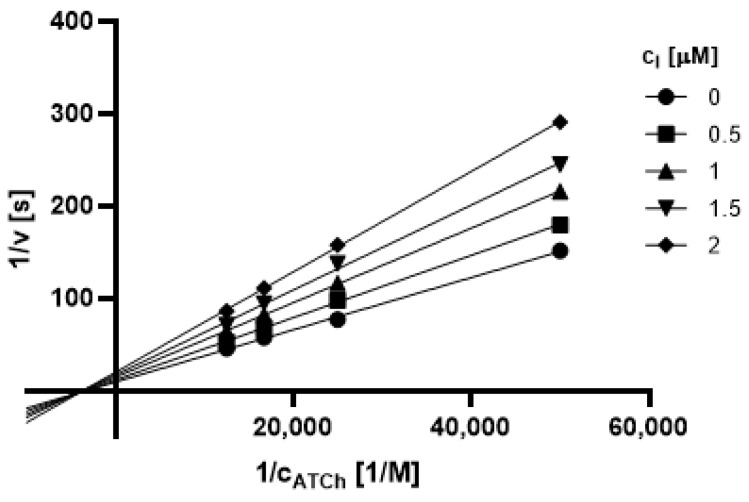
Lineweaver–Burk plot for **2a** and AChE inhibition (ATCh = acetylthiocholine).

**Figure 12:**
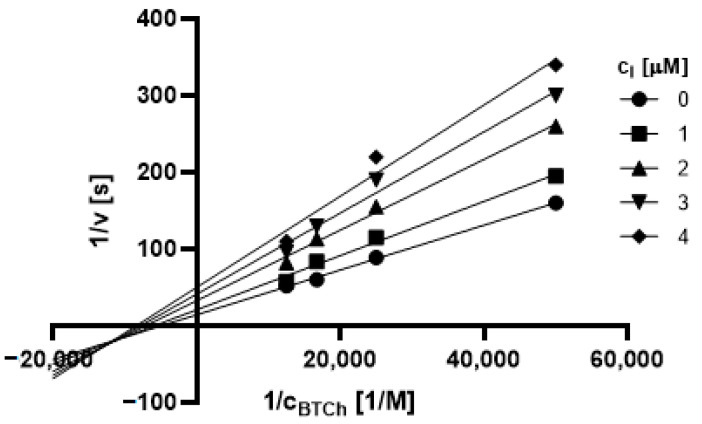
Lineweaver–Burk plot for **2a** and BuChE inhibition (BTCh = butyrylthiocholine).

**Figure 13:**
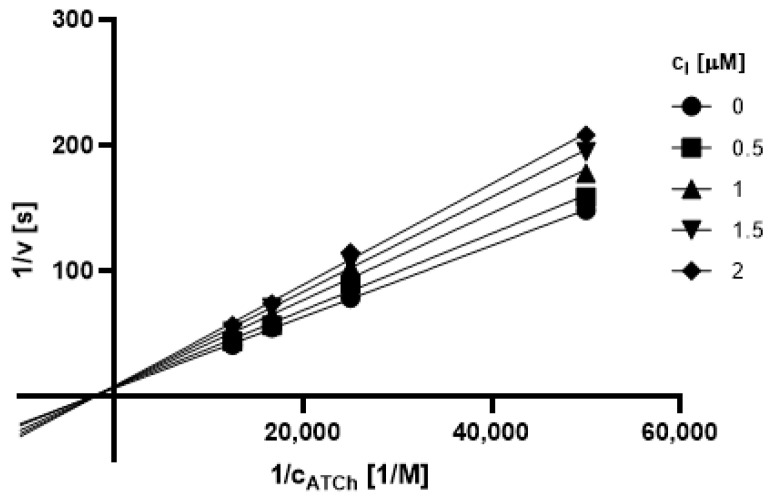
Lineweaver–Burk plot for **2e** and AChE inhibition (ATCh = acetylthiocholine).

**Figure 14:**
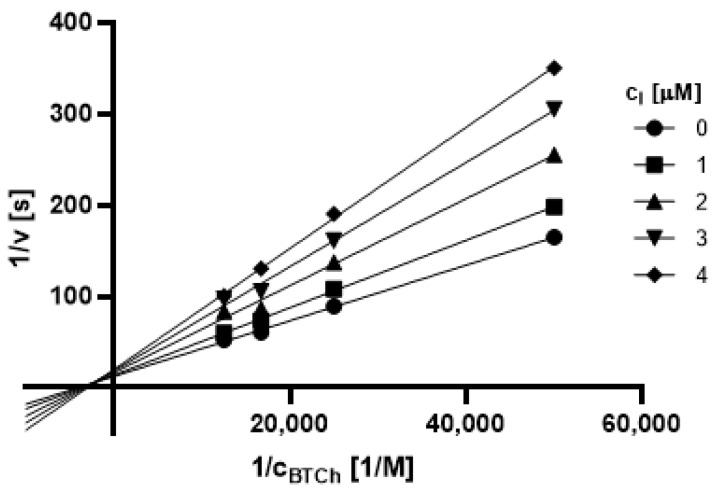
Lineweaver–Burk plot for **2e** and BuChE inhibition (BTCh = butyrylthiocholine).

The Lineweaver-Burk plots obtained for the most active dual inhibitors **1p**, **2a** and **2e** and significantly stronger AChE inhibitor **1n** are depicted in Figure 8, Figure 9, Figure 10, Figure 11, Figure 12, Figure 13 and Figure 14. Based on the plots, all these derivatives cause mixed inhibition independently on substitution patterns (5-nitrofurylidene **1n**, isatin **1p**, 3,5-diiodosalicylidene **2a** and **2e**). It is supported with changed K_M_ and V_m_ and also their ratios when compared to uninhibited reactions. There are intercepts of the lines in quadrant I, II or III in the Lineweaver–Burk plot, but not on an axis.

#### 2.3.2. Molecular Docking Study

The molecular modelling study was performed in order to estimate possible binding modes of the prepared inhibitors with ChE. It has proved to be a useful tool to elucidate the interactions between inhibitors and enzymes.

The active site of AChE was studied intensively in the past and is currently very thoroughly described. It consists of several sub-sites playing role in the stabilization of the substrate in the enzyme cavity (anionic site, Trp86, Tyr337, Phe338), contributing to the substrate specificity (oxyanion hole, acyl pocket) and securing the hydrolysis of the substrate in the first place (esteratic site, consisting of catalytic triad Ser203, His447 and Glu334) [35]. The active site is located at the bottom of strikingly narrow and deep cavity which penetrates halfway into the enzyme. At the rim of the gorge several amino acid residues (Tyr72, Asp74, Tyr124, Trp286 and Tyr341) form the so-called peripheral anionic site (PAS). Binding of the ligand to PAS can modulate the activity of AChE through conformational changes in the active site [36]. The overall structure of BuChE is very similar to that of AChE and similarly to AChE, the active site is located at the bottom of the gorge. However, a considerable amount of aromatic residues present in active center of AChE are replaced by hydrophobic residues in BuChE, thus allowing more voluminous substrates to enter [37].

The binding energies of particular ligands and enzymes are summarized in Table 4.

**Figure 15:**
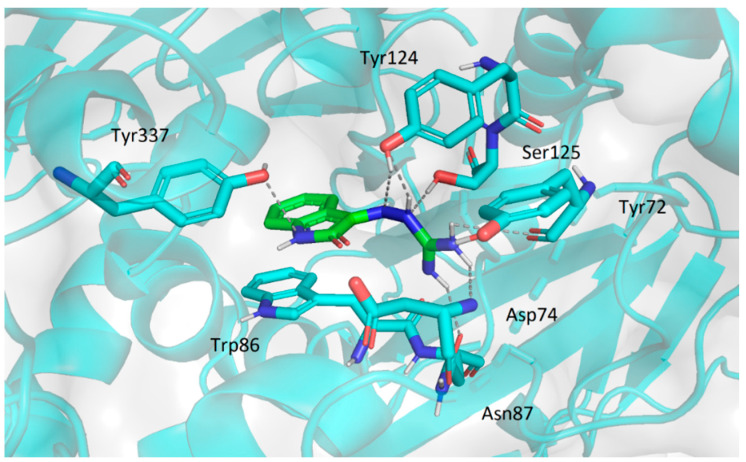
The binding mode of **1p** (green) in AChE.

**Figure 16:**
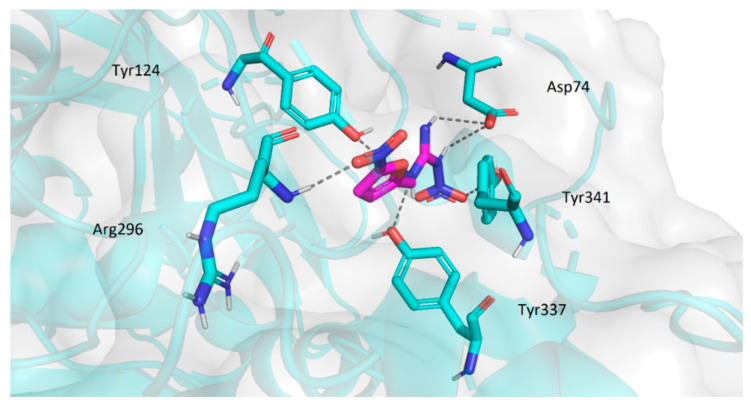
The binding mode of **3b** (magenta) in AChE.

Compound **1p**, being the most potent inhibitor of AChE in the series, was chosen as the representative for the binding mode analyses. The top-scored docking pose was observed stretched between the intrinsic anionic site and PAS. The conformation of ligand was stabilized by a number of hydrogen bond interactions (with residues Tyr72, Asp74, Asn87, Tyr124, Ser125, Tyr37) and π-π stacking (Trp86) (Figure 15). Compounds **1m**, **2a**, **2e**, and **3b** showed very similar orientation in the cavity interacting preferably with the amino acid residues of the PAS (namely Asp74, Tyr124, Trp286, Tyr341 and Tyr337; illustrated for **3b**—Figure 16).

**Figure 17:**
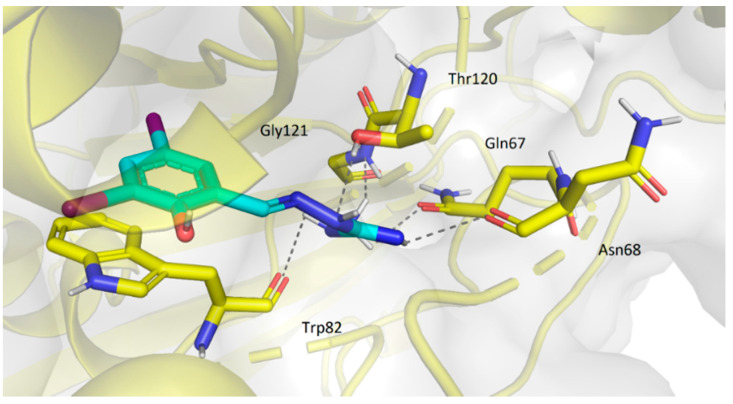
The binding mode of **2e** (cyan) in BuChE.

Concerning the interaction with BuChE, compounds **1m**, **1p**, **2a**, and **2e** showed very similar conformation near the entrance of the active gorge. Their position was stabilized by several hydrogen bonds and π-π stacking (with residues Gly121, Thr120, Gln67, Asn68 and Trp82). These interactions are demonstrated for **2e** (Figure 17).

**Figure 18:**
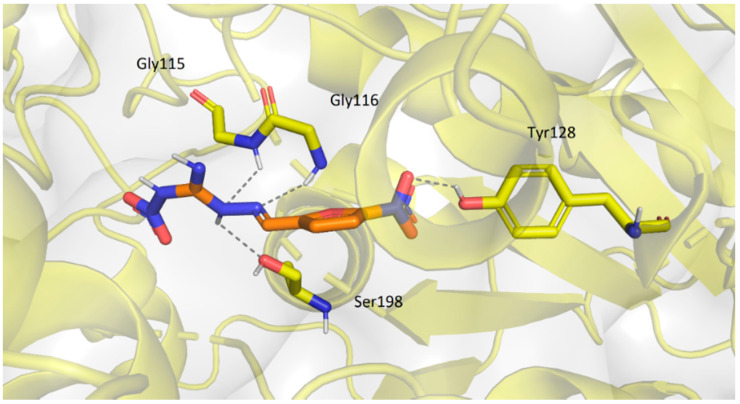
The binding mode of **3b** (orange) in BuChE.

Compound **3b** differs significantly from the other ligands in its position in the enzyme (Figure 18), which corresponds with poor in vitro results. This ligand was located deep within the gorge near the active site Ser198. Few possible hydrogen bonds stabilized the ligand in its pose (Gly115, Gly116, Tyr128, Ser198).

### 2.4. Cytotoxicity

Cytotoxicity of the tested compounds was measured using the standard hepatic cell line HepG2. The used CellTiter 96^®^ assay is based on the reduction of tetrazolium dye (MTS) in living cells to formazan, which is then determined colorimetrically. The reduction of the reagent is related to availability of NADH or NADPH. The decline in levels of these metabolically important compounds in the cell causes that the production of formazan is reduced.

The parameter IC_50_ was used as a measure of cytotoxicity, which allows the quantitative comparison of the toxicity among tested compounds (Table 5). For toxicity evaluation, we chose parent guanidine derivatives and the most promising antibacterial (**1n**, **1o**, **2c**, **2e**, and **3e**) and antifungal agents (**2e**), the most potent inhibitors of both ChE (**1m**, **1p**, **2a**, and **2e**) or only AChE (**1n**, and **2c**).

Parent amino compounds (aminoguanidine, 1,3-diaminoguanidine, nitroaminoguanidine, semicarbazide and thiosemicarbazide; data not shown) as well as the isatin derivative **1p** showed no significant cytotoxicity in the range of tested concentrations used (up to 500 µM). Other compounds can be divided into four subgroups according to the determined IC_50_ values. Three compounds showed significant toxicity with IC_50_ below 11 μM, thus being more toxic than anticancer drug tamoxifen: **1n**, **1o**, and **2c**. The second group exhibited moderate toxicity with IC_50_ higher than 29 μM and those obtained for tamoxifen: **3e**, **2a**, and **1m**. The third group containing the hydrazone **2e** showed a relatively low toxicity with IC_50_ above 100 μM. These values are comparable or superior in term of selectivity to previously reported aminoguanidine-based aromatic hydrazones [22]. The most toxic compounds exhibited similar IC_50_ values as AG-based benzothiazole hydrazones reported as apoptosis-promoting anticancer agents [15]. 

Focusing on SAR, the highest cytotoxicity was associated with the presence of (5-nitrothiophen-2-yl)methylidene, 3,5-diiodobenzylidene, 5-nitrofurfurylidene moieties attached to aminoguanidine (**1o**, **2c**, and **1n**). The replacement of thiophene by furan resulted in drop of toxicity (≈2-fold lower). Comparing 3,5-diiodobenzylidene (**2c**), 3,5-diiodosalicylidene (**1m**) and *O*-acetyl-3,5-diiodosalicylidene (**2a**) analogues, the introduction of both acetyloxy and especially hydroxy groups is beneficial in term of lower cytotoxicity. It was an unexpected finding, since salicylic phenolic group is considered to be a common cause of toxicity for eukaryotic cells and its esterification often provides fewer toxic derivatives [38]. Here, more lipophilic acetyl ester was more toxic than the derivative with free phenolic group. An introduction of amino group into 2-(2-hydroxy-3,5-diiodobenzylidene)hydrazine-1-carboximidamide (**1m**→**2e**) resulted in diminished toxicity. Similarly, the presence of thiosemicarbazide instead of AG also decreased toxicity approximately three times (**1n** vs. **3e**).

Thus, cytotoxicity is not a uniform class effect and based on aldehyde/ketone and amino compound, it is possible to modify toxic action.

To evaluate potential effect on cell membranes, we also examined identical derivatives (**1m**, **1n**, **1o**, **1p**, **2a**, **2c**, **2e**, and **3e**) using lactate dehydrogenase (LDH) release assay (CytoTox 96^®^) in HepG2 cells. It is a highly sensitive test capable of detecting early, low level damage of cell membranes. LDH is a stable cytosolic enzyme released into medium upon cell lysis. The release time from the cells to the surrounding medium is approximately 9 h. The amount of LDH released in the culture supernatants after 24 h of incubation is quantified by a combined enzyme assay that results in the conversion of the tetrazolium salt (iodonitrotetrazolium violet) to a red formazan product. This color product is determined spectrophotometrically, and its amount is proportional to the number of cells lysed, i.e., with damaged membrane integrity.

EC_50_ (half-maximal effective concentration) was used as a measure of cytotoxicity, which allows a quantitative comparison between the tested compounds. The EC_50_ of the graduated dose response curve therefore represents the concentration of compound where 50% of its maximal effect is observed (Table 6). For all tested substances, the applied toxicity parameter was found in the concentration range from 0 to 500 μM. The compounds can be divided into subgroups according to their EC_50_ values. Four compounds showed significant toxicity/membrane damaging effect with EC_50_ below 6 μM: **1n**, **1o**, **2c**, and **3e**. The second group showed milder toxicity with EC_50_ greater than 20 μM (**1m** and **2a**). The third group containing two substances (**1p**, **2e**) showed comparatively lower toxicity with EC_50_ above 100 μM.

As well as toxicity in the CellTiter 96 assay, LDH release assay also showed specific structure-dependent effect on membrane integrity that was more significant for compounds bearing 5-nitro group (**1n**, **1o**, **3e**). 

## 3. Materials and Methods

### 3.1. Chemistry

#### 3.1.1. General

All reagents and solvents were purchased from Merck (Darmstadt, Germany), Penta Chemicals (Prague, Czech Republic), Avantor (Stříbrná Skalice, Czech Republic), and Lach-Ner (Neratovice, Czech Republic). They were used as received without any purification. The reactions and the purity of the products were monitored by thin-layer chromatography (TLC) using a mixture with a ratio of dichloromethane to methanol (MeOH) and *n*-hexane of 4:1:1 (*v*/*v*/*v*) as the eluent. Plates were coated with 0.2 mm Merck 60 F254 silica gel (Merck Millipore, Darmstadt, Germany) and were visualized by UV irradiation (254 nm). The melting points were determined on a Melting Point B-560 apparatus (BÜCHI, Flawil, Switzerland) using open capillaries. The reported values are uncorrected. Infrared spectra were recorded on a FT-IR spectrometer using ATR-Ge method (Nicolet 6700 FT-IR, Thermo Fisher Scientific, Waltham, MA, USA) in the range 600–4000 cm^−1^. The NMR spectra were measured in dimethyl sulfoxide (DMSO)-*d*_6_ or deuterated chloroform (CDCl_3_) using a Varian V NMR S500 instrument (500 MHz for ^1^H and 126 MHz for ^13^C; Varian Corp., Palo Alto, CA, USA) or a JNM-ECZ 600R (600 MHz for ^1^H and 151 MHz for ^13^C; JEOL, Tokio, Japan). The chemical shifts δ are given in ppm and were referred indirectly to tetramethylsilane via signals of the solvents. The coupling constants (*J*) are reported in Hz. Elemental analysis (C, H, N) was performed on an automatic microanalyser Vario MICRO Cube Element Analyzer (Elementar Analysensysteme, Hanau, Germany). Both calculated and found values are given as percentages. 

The identity of the known compounds was established using NMR (^1^H and ^13^C) and IR spectroscopy. The characterizations are consistent with the literature data. The purity was checked by melting points measurement and elemental analysis. The compounds were considered pure if they agree within ± 0.4% with theoretical values.

#### 3.1.2. Synthesis of Precursors

Nitroaminoguanidine (*N*-nitrohydrazinecarboximidamide) was synthesized according to previously reported procedure [39]. Briefly, nitroguanidine (10 mmol) was added into 25 mL of hot water and then, hydrazine hydrate (12 mmol) was added dropwise under vigorous stirring. The reaction mixture was heated at 60 °C. After 2 h at this temperature, the reaction mixture was cooled down, acidified with concentrated hydrochloric acid and stored for 24 h at +4 °C. The resulted precipitate was filtered off, washed thoroughly by cold water and dried.

*N*-Nitrohydrazinecarboximidamide. Yellowish solid; yield 72%; mp 186.5–188 °C (186–187 °C [39]). IR (ATR): 3394, 3306, 3218, 1668, 1619, 1579, 1370, 1282, 1180, 1108, 1026, 960, 783, 672, 636 cm^−1^. ^1^H NMR (600 MHz, DMSO-*d*_6_): δ 9.28 (1H, s, NH-NO_2_), 8.22 (1H, s, NH), 7.50 (1H, s, NH), 4.63 (2H, s, NH_2_). ^13^C NMR (151 MHz, DMSO): δ 162.06. Elemental analysis for CH_5_N_5_O_2_ (119.08); calculated: C, 10.09; H, 4.23; N, 58.81, found C, 10.07; H, 4.22; N, 58.79.

2-Formyl-4,6-diiodophenyl acetate was synthesized as follows. 3,5-Diiodosalicylaldehyde (1 mmol) was dissolved in dry dichloromethane (7 mL) followed by addition of triethylamine (1.5 mmol) and after cooling to 0 °C, acetyl chloride (1.2 mol) was added in one portion under vigorous stirring. The mixture was to let stir without external cooling for one hour (based on TLC indication of a complete reaction; mobile phase: *n*-hexane: ethyl acetate 4:1, *v*/*v*). The solvent was evaporated under reduced pressure and using ethyl acetate and water, the residue was transferred into a separation funnel. The organic layer was washed with water, 10% aqueous sodium carbonate, 0.1 M aqueous hydrochloric acid, and saturated brine. The organic phase was dried over anhydrous sodium sulphate. The filtrate was concentrated under reduced pressure and *n*-hexane was added to start precipitation. After 24 h at +4 °C, the suspension was filtered off to give the ester.

2-Formyl-4,6-diiodophenyl acetate. Yellowish solid; yield 93%; mp 141.5–142.5 °C. IR (ATR): 3059, 1754, 1683, 1566, 1548, 1436, 1402, 1379, 1362, 1227, 1192, 1133, 1094, 1010, 907, 880, 733, 701, 669 cm^−1^. ^1^H NMR (600 MHz, DMSO-*d*_6_): δ 9.82 (1H, s, COH), 8.50 (1H, d, *J* = 2.1 Hz, H3), 8.16 (1H, d, *J* = 2.1 Hz, H5), 2.36 (3H, s, CH_3_). ^13^C NMR (151 MHz, DMSO): δ 189.59, 168.72, 151.76, 151.40, 140.72, 130.82, 97.52, 93.45, 21.37. Elemental analysis for C_9_H_6_I_2_O_3_ (415.95); calculated: C, 25.99; H, 1.45, found C, 25.97; H, 1.42.

3,5-Diiodobenzaldehyde was prepared from 3,5-diiodophenylmethanol [40]. The alcohol (1.5 mmol) was dissolved in 10 mL of dichloromethane and to this stirred solution, pyridinium chlorochromate (PCC; 3 mmol) was added slowly. The reaction mixture was stirred at rt for 2 h. After this time the reaction was complete (TLC indication; mobile phase: *n*-hexane: ethyl acetate 4:1, *v*/*v*). The precipitate was filtered off, while the filtrate was evaporated under reduced pressure. Using ethyl acetate, the residue was transferred into a separation funnel. The organic layer was washed with 10% aqueous sodium carbonate, and saturated brine. The organic phase was dried over anhydrous sodium sulphate. The solution was concentrated under reduced pressure and *n*-hexane was added to start precipitation. After 24 h at +4 °C, the suspension was filtered off to give the pure precursor.

3,5-Diiodobenzaldehyde. White solid; yield 99%; mp 133.5–134.5 °C (133–134 °C [40]). ^1^H NMR (600 MHz, CDCl_3_): δ 9.81 (1H, s, COH), 8.28 (1H, t, *J* = 1.6 Hz, H4), 8.13 (2H, d, *J* = 1.6 Hz, H2, H6). ^13^C NMR (151 MHz, CDCl_3_): δ 189.18, 150.65, 139.07, 137.79, 95.37. Elemental analysis for C_7_H_4_I_2_O (357.92); calculated: C, 23.49; H, 1.13, found C, 23.52; H, 1.12.

#### 3.1.3. Synthesis of Targeted Derivatives **1**–**3**

Hydrazones: Aminoguanidine hydrochloride (or nitroaminoguanidine, 1,3-diaminoguanidine hydrochloride, thiosemicarbazide, or semicarbazide hydrochloride; 1 mmol) was dissolved in 8–10 mL of MeOH and then an appropriate aldehyde or ketone (1.1 mmol or 2.2 mmol for the synthesis of disubstituted 1,3-diaminoguanidines **2g** and **3c**) was added in one portion. The reaction mixture was heated under reflux for 3 h and then let to stir to cool down to room temperature overnight. Then, the reaction mixture was stored at −20 °C for 2 h. If there was present a precipitate, it was collected by filtration. The isolated crystals were suspended in 5% sodium bicarbonate and stirred for 30 min. Then, the suspension was filtered off, the crystals were washed by cold water, diethyl ether and dried. When no spontaneous precipitation was observed, the methanolic solution was diluted by 5% sodium bicarbonate, stored for 1 h at +4 °C. In some cases, the reaction mixture was partly evaporated after basification (e.g., **2a** and **3a**). The resulting precipitate was collected by filtration, washed by cold water, diethyl ether and dried. The products were crystallized from methanol if necessary. 

The imine **3a** was prepared in water under reflux for 6 h and the solution was basified using solid sodium carbonate.

Synthesis of the reduced analogue **2d**: Aminoguanidine hydrochloride (1 mmol) was dissolved in 10 mL of methanol and then 3,5-diiodosalicylaldehyde (1.1 mmol, 411.3 mg) was added in one portion. After a complete dissolution, sodium cyanoborohydride (100 mg; 1.6 mmol) was added. The mixture was stirred for 48 h at room temperature. Then, the reaction mixture was diluted with 5% sodium bicarbonate and let to stir over night at room temperature. The precipitate was filtered off, washed by water, and dried to obtain pure **2d**.

(*E*)-2-Benzylidenehydrazine-1-carboximidamide **1a**. White solid; yield 78%; mp 178–181 °C (180–182 °C [13]). IR (ATR): 3460, 3282, 3026, 1699, 1630, 1601, 1501, 1447, 1394, 1366, 1229, 1177, 1123, 1013, 968, 942, 829, 753, 709, 684, 642, 627 cm^−1^. ^1^H NMR (600 MHz, DMSO-*d*_6_): δ 7.96 (1H, s, CH = N), 7.63 (2H, d, *J* = 7.6 Hz, H2, H6), 7.29 (2H, t, *J* = 7.6 Hz, H3, H5), 7.21 (1H, t, *J* = 7.7 Hz, H4), 5.97 (2H, s, NH_2_), 5.62 (2H, s, NH_2_). ^13^C NMR (151 MHz, DMSO): δ 160.91, 143.86, 137.36, 128.89, 128.34, 126.79. Elemental analysis for C_8_H_10_N_4_ (162.20); calculated: C, 59.24; H, 6.21; N, 34.54, found C, 59.21; H, 6.20; N, 34.56.

(*E*)-2-(2-Chlorobenzylidene)hydrazine-1-carboximidamide **1b**. White solid; yield 81%; mp 160–162 °C (145–146 °C [13]). IR (ATR): 3455, 3019, 1695, 12627, 1596, 1495, 1393, 1355, 1280, 1224, 1173, 1150, 1109, 1051, 1012, 937, 831, 751, 706, 638, 617 cm^−1^. ^1^H NMR (600 MHz, DMSO-*d*_6_): δ 8.23 (1H, s, CH = N), 8.09 (1H, dd, *J* = 7.5, 2.1 Hz, H6), 7.35 (1H, dd, *J* = 7.6, 1.6 Hz, H3), 7.25–7.19 (2H, m, H4, H5), 6.00 (2H, s, NH_2_), 5.62 (2H, s, NH_2_). ^13^C NMR (151 MHz, DMSO): δ 161.82, 138.93, 134.45, 132.03, 130.04, 129.36, 127.49, 127.29. Elemental analysis for C_8_H_9_ClN_4_ (196.64); calculated: C, 48.87; H, 4.61; N, 28.49, found C, 48.85; H, 4.57; N, 28.53.

(*E*)-2-(3-Chlorobenzylidene)hydrazine-1-carboximidamide **1c**. White solid; yield 95%; mp 166–168 °C (164–166 °C [13]). IR (ATR): 3463, 3008, 1699, 1635, 1593, 1477, 1407, 1358, 1288, 1235, 1180, 1128, 1097, 1071, 1016, 940, 893, 821, 783, 729, 712, 680, 656, 630 cm^−1^. ^1^H NMR (600 MHz, DMSO-*d*_6_): δ 7.91 (1H, s, CH = N), 7.76 (1H, t, *J* = 2.0 Hz, H2), 7.52 (1H, dd, *J* = 7.7, 2.0 Hz, H6), 7.29 (1H, t, *J* = 7.8 Hz, H5), 7.24–7.21 (1H, m, H4), 6.00 (2H, s, NH_2_), 5.55 (2H, s, NH_2_). ^13^C NMR (151 MHz, DMSO): δ 161.60, 141.84, 139.97, 133.91, 130.67, 127.68, 125.73, 125.53. Elemental analysis for C_8_H_9_ClN_4_ (196.64); calculated: C, 48.87; H, 4.61; N, 28.49, found C, 48.86; H, 4.60; N, 28.52.

(*E*)-2-(4-Chlorobenzylidene)hydrazine-1-carboximidamide **1d**. Yellow solid; yield 96%; mp 220.5–222 °C (206–208 °C [13]). IR (ATR): 3471, 3366, 3028, 1704, 1640, 1603, 1584, 1544, 1441, 1409, 1348, 1308, 1243, 1229, 1156, 1102, 941, 887, 860, 766, 752, 665, 645 cm^−1^. ^1^H NMR (600 MHz, DMSO-*d*_6_): δ 7.93 (1H, s, CH = N), 7.67–7.63 (2H, m, H2, H6), 7.34–7.31 (2H, m, H3, H5), 5.94 (2H, s, NH_2_), 5.54 (2H, s, NH_2_). ^13^C NMR (151 MHz, DMSO): δ 161.32, 142.23, 136.53, 132.40, 128.88, 128.29. Elemental analysis for C_8_H_9_ClN_4_ (196.64); calculated: C, 48.87; H, 4.61; N, 28.49, found C, 48.91; H, 4.65; N, 28.46.

(*E*)-2-(3-Chloro-2-hydroxybenzylidene)hydrazine-1-carboximidamide **1e**. Yellowish solid; yield 99%; mp 205.5–206.5 °C. IR (ATR): 3448, 3366, 3068, 1674, 1619, 1593, 1545, 1485, 1450, 1409, 1339, 1298, 1273, 1234, 1194, 1174, 1144, 1070, 921, 834, 774, 766, 726, 694, 640 cm^−1^. ^1^H NMR (500 MHz, DMSO-*d*_6_): δ 12.41 (1H, s, OH), 8.22 (1H, s, CH = N), 7.31 (1H, dd, *J* = 7.7, 1.6 Hz, H6), 7.26 (1H, dd, *J* = 7.6, 1.6 Hz, H4), 6.79 (1H, t, *J* = 7.7 Hz, H5), 6.01 (2H, s, NH_2_), 5.86 (2H, s, NH_2_). ^13^C NMR (126 MHz, DMSO): δ 159.34, 146.64, 129.08, 128.06, 124.38, 121.68, 119.98, 118.95. Elemental analysis for C_8_H_9_ClN_4_O (212.64); calculated: C, 45.19; H, 4.27; N, 26.35, found C, 45.23; H, 4.26; N, 26.33.

(*E*)-2-(4-Chloro-2-hydroxybenzylidene)hydrazine-1-carboximidamide [41] **1f**. White solid; yield 88%; mp 182–184.5 °C. IR (ATR): 3454, 3348, 3050, 1697, 1625, 1595, 1494, 1388, 1355, 1280, 1221, 1174, 1115, 1050, 1011, 958, 916, 880, 830, 807, 750, 706, 659, 645, 623 cm^−1^. ^1^H NMR (600 MHz, DMSO-*d*_6_): δ 11.66 (1H, s, OH), 8.13 (1H, s, CH = N), 7.37 (1H, d, *J* = 8.2 Hz, H6), 6.85 (1H, d, *J* = 2.1 Hz, H3), 6.83 (1H, dd, *J* = 8.2, 2.1 Hz, H5), 5.76 (2H, s, NH_2_), 5.56 (2H, s, NH_2_). ^13^C NMR (151 MHz, DMSO): δ 160.05, 158.26, 145.75, 133.08, 130.65, 120.35, 119.50, 115.93. Elemental analysis for C_8_H_9_ClN_4_O (212.64); calculated: C, 45.19; H, 4.27; N, 26.35, found C, 45.21; H, 4.30; N, 26.38.

(*E*)-2-(5-Chloro-2-hydroxybenzylidene)hydrazine-1-carboximidamide **1g**. White solid; yield 94%; mp 164.5–165.5 °C (167–169 °C [41]). IR (ATR): 3439, 3340, 3058, 1701, 1631, 1605, 1557, 1480, 1420, 1394, 1366, 1278, 1257, 1242, 1189, 1107, 1026, 942, 916, 870, 829, 819, 784, 710, 658, 646, 630 cm^−1^. ^1^H NMR (600 MHz, DMSO-*d*_6_): δ 11.26 (1H, s, OH), 8.12 (1H, s, CH = N), 7.47 (1H, d, *J* = 2.7 Hz, H6), 7.08 (1H, dd, *J* = 8.6, 2.6 Hz, H4), 6.80 (1H, d, *J* = 8.7 Hz, H3), 5.81 (2H, s, NH_2_), 5.58 (2H, s, NH_2_). ^13^C NMR (151 MHz, DMSO): δ 160.32, 155.93, 144.72, 128.62, 128.10, 123.08, 123.06, 117.83. Elemental analysis for C_8_H_9_ClN_4_O (212.64); calculated: C, 45.19; H, 4.27; N, 26.35, found C, 45.19; H, 4.29; N, 26.33.

(*E*)-2-(6-Chloro-2-hydroxybenzylidene)hydrazine-1-carboximidamide **1h**. White solid; yield 97%; mp 193.5–196 °C. IR (ATR): 3464, 3292, 3009, 1704, 1632, 1601, 1573, 1501, 1386, 1348, 1306, 1265, 1210, 1171, 1115, 1013, 945, 918, 840, 828, 774, 731, 702, 634 cm^−1^. ^1^H NMR (500 MHz, DMSO-*d*_6_): δ 12.50 (1H, s, OH), 8.48 (1H, s, CH = N), 7.13 (1H, t, *J* = 8.1 Hz, H4), 6.92 (1H, dd, *J* = 8.0, 1.1 Hz, H3), 6.83 (1H, dd, *J* = 8.0, 1.1 Hz, H5), 5.87 (2H, s, NH_2_), 5.75 (2H, s, NH_2_). ^13^C NMR (126 MHz, DMSO): δ 159.99, 158.91, 143.48, 132.06, 129.68, 120.02, 117.06, 115.23. Elemental analysis for C_8_H_9_ClN_4_O (212.64); calculated: C, 45.19; H, 4.27; N, 26.35, found C, 45.15; H, 4.24; N, 26.37.

(*E*)-2-(2-Hydroxy-5-iodobenzylidene)hydrazine-1-carboximidamide **1i**. White solid; yield 98%; mp 171–172 °C. IR (ATR): 3432, 3079, 1654, 1639, 1605, 1579, 1544, 1475, 1456, 1431, 1405, 1358, 1284, 1263, 1239, 1185, 1122, 1085, 952, 889, 826, 783, 742, 7025, 688, 619 cm^−1^. ^1^H NMR (600 MHz, DMSO-*d*_6_): δ 11.35 (1H, s, OH), 8.10 (1H, s, CH = N), 7.70 (1H, d, *J* = 2.4 Hz, H6), 7.34 (1H, dd, *J* = 8.5, 2.3 Hz, H4), 6.63 (1H, d, *J* = 8.6 Hz, H3), 5.57 (2H, s, NH_2_), 5.55 (2H, s, NH_2_). ^13^C NMR (151 MHz, DMSO): δ 160.23, 157.14, 144.89, 137.28, 136.92, 124.18, 118.85, 81.30. Elemental analysis for C_8_H_9_IN_4_O (304.09); calculated: C, 31.60; H, 2.98; N, 18.42, found C, 31.65; H, 3.02; N, 18.40.

(*E*)-2-(3,5-Dichloro-2-hydroxybenzylidene)hydrazine-1-carboximidamide [12] **1j**. Yellowish solid; yield 99%; mp 202.5–203.5 °C. IR (ATR): 3472, 3339, 3025, 1701, 1635, 1557, 1491, 1397, 1359, 1299, 1240, 1175, 1127, 1098, 1088, 1013, 950, 930, 831, 818, 784, 710, 702, 679, 639 cm^−1^. ^1^H NMR (600 MHz, DMSO-*d*_6_): δ 12.60 (1H, s, OH), 8.37 (1H, s, CH = N), 7.61 (1H, d, *J* = 2.7 Hz, H6), 7.22 (1H, d, *J* = 2.8 Hz, H4), 7.13 (4H, s, NH_2_). ^13^C NMR (151 MHz, DMSO): δ 157.75, 157.42, 145.29, 145.22, 129.44, 129.38, 125.39, 122.46. Elemental analysis for C_8_H_8_Cl_2_N_4_O (247.08); calculated: C, 38.89; H, 3.26; N, 22.68, found C, 38.88; H, 3.26; N, 22.67.

(*E*)-2-(3-Bromo-5-chloro-2-hydroxybenzylidene)hydrazine-1-carboximidamide **1k**. Yellow solid; yield 95%; mp 208–209 °C. IR (ATR): 3471, 3068, 1677, 1634, 1430, 1401, 1343, 1307, 1241, 1226, 1155, 1016, 946, 932, 888, 866, 851, 749, 731, 702, 669, 635 cm^−1^. ^1^H NMR (600 MHz, DMSO-*d*_6_): δ 12.52 (1H, s, OH), 8.33 (1H, s, CH = N), 7.65 (1H, d, *J* = 2.7 Hz, H6), 7.36 (1H, d, *J* = 2.7 Hz, H4), 7.06 (4H, s, NH_2_). ^13^C NMR (151 MHz, DMSO): δ 157.72, 157.41, 145.30, 132.16, 126.49, 126.03, 122.28, 81.96. Elemental analysis for C_8_H_8_BrClN_4_O (291.53); calculated: C, 32.96; H, 2.77; N, 19.22, found C, 33.01; H, 2.74; N, 19.26.

(*E*)-2-(5-Chloro-2-hydroxy-3-iodobenzylidene)hydrazine-1-carboximidamide **1l**. Yellow solid; yield 96%; mp 202–203 °C. IR (ATR): 3470, 3441, 3366, 3345, 3074, 1621, 1600, 1570, 1549, 1442, 1424, 1337, 1262, 1222, 1184, 1164, 1102, 978, 940, 867, 823, 737, 708, 688, 632 cm^−1^. ^1^H NMR (600 MHz, DMSO-*d*_6_): δ 12.57 (1H, s, OH), 8.21 (1H, s, CH = N), 7.61 (1H, d, *J* = 2.7 Hz, H6), 7.53 (1H, d, *J* = 2.7 Hz, H4), 6.70 (4H, s, NH_2_). ^13^C NMR (151 MHz, DMSO): δ 158.35, 148.89, 145.53, 137.41, 127.74, 127.52, 120.93, 87.86. Elemental analysis for C_8_H_8_ClIN_4_O (338.53); calculated: C, 28.38; H, 2.38; N, 16.55, found C, 22.42; H, 2.39; N, 16.58.

(*E*)-2-(2-Hydroxy-3,5-diiodobenzylidene)hydrazine-1-carboximidamide **1m**. Yellow solid; yield 96%; mp 195–196.5 °C. IR (ATR): 3443, 3419, 3350, 3064, 1674, 1630, 1595, 1562, 1429, 1401, 1333, 1284, 1265, 1244, 1226, 1156, 1051, 1011, 963, 927, 874, 865, 841, 754, 734, 704, 665, 645, 527 cm^−1^. ^1^H NMR (600 MHz, DMSO-*d*_6_): δ 12.05 (1H, s, OH), 8.30 (1H, s, CH = N), 8.14 (1H, d, *J* = 2.2 Hz, H6), 8.02 (1H, d, *J* = 2.1 Hz, H4), 6.78 (4H, s, NH_2_). ^13^C NMR (151 MHz, DMSO): δ 155.77, 155.59, 147.80, 144.70, 137.01, 123.66, 91.25, 84.72. Elemental analysis for C_8_H_8_I_2_N_4_O (429.99); calculated: C, 22.35; H, 1.88; N, 13.03, found C, 22.31; H, 1.89; N, 13.06.

(*E*)-2-[(5-Nitrofuran-2-yl)methylene]hydrazine-1-carboximidamide **1n**. Red solid; yield 90%; mp 236–236.5 °C. IR (ATR): 3447, 3429, 3315, 3063, 1658, 1615, 1558, 1484, 1459, 1381, 1354, 1304, 1233, 1179, 1133, 1024, 980, 959, 919, 820, 811, 749, 732, 725, 670, 613 cm^−1^. ^1^H NMR (600 MHz, DMSO-*d*_6_): δ 7.80 (1H, s, CH = N), 7.71 (1H, d, *J* = 4.0 Hz, H4), 7.00 (1H, d, *J* = 4.1 Hz, H3), 6.16 (4H, s, NH_2_). ^13^C NMR (151 MHz, DMSO): δ 162.86, 157.39, 151.09, 129.93, 116.93, 110.32. Elemental analysis for C_6_H_7_N_5_O_3_ (197.15); calculated: C, 36.55; H, 3.58; N, 35.52, found C, 36.51; H, 3.60; N, 35.53.

(*E*)-2-[(5-Nitrothiophen-2-yl)methylene]hydrazine-1-carboximidamide **1o**. Red-brown solid; yield 93%; mp 223–225 °C. IR (ATR): 3391, 3080, 1652, 1530, 1483, 1436, 1411, 1368, 1339, 1308, 1286, 1224, 1161, 1100, 1046, 1019, 929, 910, 824, 817, 775, 734, 708, 690 cm^−1^. ^1^H NMR (500 MHz, DMSO-*d*_6_): δ 8.08 (1H, s, CH = N), 8.01 (1H, d, *J* = 4.4 Hz, H4), 7.15 (1H, d, *J* = 4.4 Hz, H3), 6.11 (4H, s, NH_2_). ^13^C NMR (126 MHz, DMSO): δ 162.02, 152.54, 147.55, 135.36, 131.26, 124.81. Elemental analysis for C_6_H_7_N_5_O_2_S (213.22); calculated: C, 33.80; H, 3.31; N, 32.85, found C, 33.76; H, 3.33; N, 32.89.

2-(2-Oxoindolin-3-ylidene)hydrazine-1-carboximidamide **1p**. Yellow solid; yield 65%; mp >250 °C (247–249 °C [42]). IR (ATR): 3399, 3350, 3174, 1678, 1622, 1594, 1490, 1470, 1371, 1336, 1273, 1214, 1149, 1106, 1050, 1033, 1011, 930, 889, 835, 789, 749, 705, 677, 650, 616 cm^−1^. ^1^H NMR (500 MHz, DMSO-*d*_6_): δ 10.16 (1H, s, amide N-H), 7.57 (1H, dd, *J* = 7.5, 1.2 Hz, H4), 7.09 (1H, td, *J* = 8.0, 1.4 Hz, H6), 6.87 (1H, td, *J* = 7.5, 1.3 Hz, H5), 6.80–6.71 (5H, m, H7, NH_2_). ^13^C NMR (126 MHz, DMSO): δ 164.88, 158.90, 139.77, 132.24, 127.24, 124.53, 120.65, 118.65, 109.09. Elemental analysis for C_9_H_9_N_5_O (203.21); calculated: C, 53.20; H, 4.46; N, 34.47, found C, 53.23; H, 4.42; N, 34.50.

(*E*)-2-[(2-Carbamimidoylhydrazineylidene)methyl]-4,6-diiodophenyl acetate **2a**. Yellow solid; yield 74%; mp 160–162 °C. IR (ATR): 3400, 1761, 1702, 1684, 1623, 1559, 1544, 1517, 1507, 1490, 1458, 1424, 1339, 1244, 1186, 1130, 1014, 930, 894, 864, 741, 698, 643, 632, 626 cm^−1^. ^1^H NMR (600 MHz, DMSO-*d*_6_): δ 8.44 (1H, d, *J* = 2.0 Hz, H3), 8.09 (1H, d, *J* = 2.0 Hz, H5), 7.88 (1H, s, CH = N), 6.40 (4H, s, NH_2_), 2.35 (3H, s, CH_3_). ^13^C NMR (151 MHz, DMSO): δ 168.75, 159.57, 149.16, 145.90, 137.23, 134.86, 131.85, 95.67, 93.52, 21.27. Elemental analysis for C_10_H_10_I_2_N_4_O_2_ (472.02); calculated: C, 25.45; H, 2.14; N, 11.87, found C, 25.47; H, 2.12; N, 11.90.

(*E*)-2-(4-Hydroxy-3,5-diiodobenzylidene)hydrazine-1-carboximidamide **2b**. Greyish solid; yield 91%; mp 197–198.5 °C. IR (ATR): 3445, 3137, 1673, 1630, 1606, 1569, 1439, 1395, 1348, 1316, 1260, 1219, 1169, 1005, 962, 943, 880, 832, 741, 704, 639 cm^−1^. ^1^H NMR (600 MHz, DMSO-*d*_6_): δ 10.84 (1H, s, OH), 7.92 (2H, s, H2, H6), 7.72 (1H, s, CH = N), 7.59 (2H, s, NH_2_), 7.06 (2H, s, NH_2_). ^13^C NMR (151 MHz, DMSO): δ 156.223, 155.20, 146.74, 138.04, 130.66, 90.97. Elemental analysis for C_8_H_8_I_2_N_4_O (429.99); calculated: C, 22.35; H, 1.88; N, 13.03, found C, 22.36; H, 1.91; N, 13.00.

(*E*)-2-(3,5-Diiodobenzylidene)hydrazine-1-carboximidamide **2c**. White solid; yield 80%; mp 209–211 °C. IR (ATR): 3465, 3156, 1677, 1632, 1570, 1534, 1421, 1401, 1348, 1224, 1159, 1117, 1091, 1015, 991, 957, 939, 903, 848, 783, 711, 670, 623 cm^−1^. ^1^H NMR (600 MHz, DMSO-*d*_6_): δ 8.01 (2H, s, H2, H6), 7.84 (1H, s, H4), 7.78 (1H, s, CH = N), 6.05 (2H, s, NH_2_), 5.56 (2H, s, NH_2_). ^13^C NMR (151 MHz, DMSO): δ 161.85, 143.05, 141.95, 140.09, 134.17, 96.71. Elemental analysis for C_8_H_8_I_2_N_4_ (413.99); calculated: C, 23.21; H, 1.95; N, 13.53, found C, 23.17; H, 1.92; N, 13.49.

2-(2-Hydroxy-3,5-diiodobenzyl)hydrazine-1-carboximidamide **2d**. White solid; yield 82%; mp 203–204 °C. IR (ATR): 3452, 3349, 1669, 1650, 1630, 1443, 1345, 1280, 1243, 1229, 1154, 1099, 1075, 1020, 1004, 931, 885, 861, 852, 819, 754, 695, 649 cm^−1^. ^1^H NMR (600 MHz, DMSO-*d*_6_): δ 7.63 (1H, d, *J* = 2.2 Hz, H6), 7.13 (1H, d, *J* = 2.1 Hz, H4), 7.03 (3H, s, NH_2_), 4.64–4.35 (4H, m, NH-NH, CH_2_). ^13^C NMR (151 MHz, DMSO): δ 159.32, 156.71, 142.86, 133.38, 130.67, 89.52, 78.69, 61.19. Elemental analysis for C_8_H_10_I_2_N_4_O (432.00); calculated: C, 22.24; H, 2.33; N, 12.97, found C, 22.27; H, 2.33; N, 13.01.

(*E*)-Hydrazineyl[2-(2-hydroxy-3,5-diiodobenzylidene)hydrazineyl]methaniminium chloride **2e**. White solid; yield 90%; mp 236–238 °C. IR (ATR): 3397, 3324, 3207, 1697, 1644, 1595, 1454, 1345, 1282, 1266, 1231, 1154, 990, 964, 942, 858, 733, 669, 640, 630 cm^−1^. ^1^H NMR (600 MHz, DMSO-*d*_6_): δ 11.98 (1H, s, OH), 10.06 (1H, s, NH), 9.47 (1H, s, NH), 8.52 (1H, s, CH = N), 8.06 (1H, d, *J* = 2.1 Hz, H6), 8.02 (1H, d, *J* = 2.1 Hz, H4), 7.96 (2H, s, NH_2_), 4.87 (2H, s, N-NH_2_). ^13^C NMR (151 MHz, DMSO): δ 156.70, 155.84, 155.52, 147.70, 137.08, 123.73, 91.22, 84.73. Elemental analysis for C_8_H_10_ClI_2_N_5_O (481.46); calculated: C, 19.96; H, 2.09; N, 14.55, found C, 20.01; H, 2.12; N, 14.52.

(*E*)-2-(2-Hydroxy-3,5-diiodobenzylidene)-*N*-nitrohydrazine-1-carboximidamide **2f**. White solid; yield 99%; mp > 250 °C. IR (ATR): 3413, 3221, 1638, 1598, 1578, 1417, 1384, 1363, 1340, 1280, 1231, 1160, 1061, 953, 919, 868, 735, 721, 680, 659, 607 cm^−1^. ^1^H NMR (600 MHz, DMSO-*d*_6_): δ 11.80 (1H, s, OH), 9.96 (1H, s, NH-NO_2_), 8.84 (2H, s, NH), 8.26 (1H, s, CH = N), 8.14 (1H, d, *J* = 2.2 Hz, H6), 8.02 (1H, d, *J* = 2.1 Hz, H4). ^13^C NMR (151 MHz, DMSO): δ 158.18, 155.47, 147.67, 144.95, 137.24, 123.97, 91.04, 84.72. Elemental analysis for C_8_H_7_I_2_N_5_O_3_ (474.98); calculated: C, 20.23; H, 1.49; N, 14.74, found C, 20.26; H, 1.49; N, 14.70.

Bis[2 -((*E*)-2-hydroxy-3,5-diiodobenzylidene)hydrazineyl]methaniminium chloride **2g**. Yellow solid; yield 50%; mp 234–236 °C. IR (ATR): 3338, 1643, 1625, 1554, 1428, 1368, 1349, 1282, 1264, 1226, 1162, 1080, 958, 940, 925, 863, 734, 655, 641, 625 cm^−1^. ^1^H NMR (600 MHz, DMSO-*d*_6_): δ 11.18 (4H, broad s, OH, N = NH), 8.34 (2H, s, CH = N), 8.05–7.98 (4H, m, H4, H6), 7.86 (2H, s, NH_2_). ^13^C NMR (151 MHz, DMSO): δ 155.88, 147.09, 146.96, 145.74, 137.43, 123.55, 90.06, 84.12. Elemental analysis for C_15_H_12_ClI_4_N_5_O_2_ (837.36); calculated: C, 21.52; H, 1.44; N, 8.36, found C, 21.55; H, 1.43; N, 8.38.

(*E*)-2-(2-Hydroxy-3,5-diiodobenzylidene)hydrazine-1-carboxamide [43] **2h**. White solid; yield 93%; mp >250 °C. IR (ATR): 3441, 3378, 3054, 2895, 1668, 1588, 1437, 1415, 1338, 1270, 1228, 1162, 1113, 940, 908, 863, 735, 665, 625 cm^−1^. ^1^H NMR (600 MHz, DMSO-*d*_6_): δ 11.10 (1H, s, OH), 10.41 (1H, s, NNH), 7.93 (1H, s, CH = N), 7.92 (1H, d, *J* = 2.1 Hz, H6), 7.83 (1H, d, *J* = 2.1 Hz, H4), 6.45 (2H, s, CONH_2_). ^13^C NMR (151 MHz, DMSO): δ 156.14, 155.59, 146.02, 139.45, 137.52, 123.17, 89.21, 83.50. Elemental analysis for C_8_H_7_I_2_N_3_O_2_ (430.97); calculated: C, 22.30; H, 1.64; N, 9.75, found C, 22.32; H, 1.66; N, 9.76.

(*E*)-2-(2-Hydroxy-3,5-diiodobenzylidene)hydrazine-1-carbothioamide [44] **2i**. White solid; yield 84%; mp > 250 °C. IR (ATR): 3442, 3293, 3170, 3032, 1612, 1591, 1539, 1469, 1424, 1356, 1284, 1267, 1230, 1163, 1060, 938, 863, 831, 734, 690, 663, 655, 605 cm^−1^. ^1^H NMR (600 MHz, DMSO-*d*_6_): δ 11.45 (1H, s, OH), 9.91 (1H, s, NNH), 8.20–8.15 (3H, m, CH = N, H6, CS-NH_A_), 8.02 (1H, s, CS-NH_B_), 7.97 (1H, d, *J* = 2.1 Hz, H4). ^13^C NMR (151 MHz, DMSO): δ 178.45, 155.48, 147.00, 140.89, 137.14, 124.30, 90.70, 84.63. Elemental analysis for C_8_H_7_I_2_N_3_OS (447.03); calculated: C, 21.49; H, 1.58; N, 9.40, found C, 21.51; H, 1.59; N, 9.36.

(*E*)-2-(Furan-2-ylmethylene)hydrazine-1-carboximidamide **3a**. Brownish solid; yield 97%; mp 162–165 °C (175–178 °C [13]). IR (ATR): 3446, 3292, 3116, 1637, 1606, 1535, 1490, 1385, 1336, 1159, 1137, 1013, 1004, 983, 942, 913, 887, 808, 783, 740, 731, 724, 662 cm^−1^. ^1^H NMR (600 MHz, DMSO-*d*_6_): δ 7.81 (1H, s, CH = N), 7.60 (1H, d, *J* = 1.6 Hz, H5), 6.57 (1H, d, *J* = 3.3 Hz, H3), 6.49–6.47 (1H, m, H4), 5.76 (2H, s, NH_2_), 5.50 (2H, s, NH_2_). ^13^C NMR (151 MHz, DMSO): δ 161.01, 152.72, 143.26, 134.06, 112.23, 108.89. Elemental analysis for C_6_H_8_N_4_O (152.16); calculated: C, 31.60; H, 2.98; N, 18.42, found C, 31.65; H, 3.02; N, 18.40.

(*E*)-*N*-Nitro-2-[(5-nitrofuran-2-yl)methylene]hydrazine-1-carboximidamide **3b**. Yellow solid; yield 96%; mp 247–249 °C (249–250 °C [26]). IR (ATR): 3386, 3147, 1652, 1565, 1510, 1486, 1439, 1381, 1353, 1316, 1280, 1224, 1182, 1150, 1075, 1025, 956, 927, 827, 818, 738, 645 cm^−1^. ^1^H NMR (600 MHz, DMSO-*d*_6_): δ 12.13 (1H, s, NH-NO_2_), 8.93 (1H, s, NH), 8.45 (1H, s, NH), 8.04 (1H, s, CH = N), 7.77 (1H, d, *J* = 3.9 Hz, H4), 7.40 (1H, d, *J* = 4.0 Hz, H3). ^13^C NMR (151 MHz, DMSO): δ 158.25, 152.35, 152.21, 135.01, 115.41, 115.04. Elemental analysis for C_6_H_6_N_6_O_5_ (242.15); calculated: C, 29.76; H, 2.50; N, 34.71, found C, 29.78; H, 2.49; N, 34.68.

(*E*)-2-[(5-Nitrofuran-2-yl)methylene]-*N*’-[(*E*)-(5-nitrofuran-2-yl)methylene]hydrazine-1-carboximidamide hydrochloride **3c**. Orange-red solid; yield 93%; mp >250 °C (275–285 °C decomp. [45]). IR (ATR): 3389, 3149, 3123, 1666, 1624, 1600, 1564, 1524, 1508, 1481, 1386, 1357, 1326, 1311, 1265, 1251, 1205, 1181, 1148, 1108, 1035, 1022, 964, 946, 829, 819, 780, 737 cm^−1^. ^1^H NMR (600 MHz, DMSO-*d*_6_): δ 13.02 (2H, s, NH-N=), 8.68 (2H, s, NH_2_), 8.51 (2H, s, CH = N), 7.81 (2H, d, *J* = 3.9 Hz, H4), 7.53 (2H, d, *J* = 4.0 Hz, H3). ^13^C NMR (151 MHz, DMSO): δ 153.43, 152.58, 151.67, 137.79, 116.18, 115.30. Elemental analysis for C_11_H_10_ClN_7_O_6_ (371.69); calculated: C, 35.55; H, 2.71; N, 26.38, found C, 35.51; H, 2.66; N, 26.40.

(*E*)-2-[(5-Nitrofuran-2-yl)methylene]hydrazine-1-carboxamide **3d**. Yellow solid; yield 73%; mp >250 °C (240–244 °C [46]). IR (ATR): 3458, 3241, 3136, 1707, 1582, 1503, 1457, 1390, 1348, 1327, 314, 1251, 1199, 1150, 1020, 969, 903, 820, 807, 786, 760, 737 649 cm^−1^. ^1^H NMR (600 MHz, DMSO-*d*_6_): δ 10.78 (1H, s, CONHN), 7.75–7.73 (2H, m, CH = N, H4), 7.16 (1H, d, *J* = 4.0 Hz, H3), 6.53 (2H, s, CONH_2_). ^13^C NMR (151 MHz, DMSO): δ 157.02, 154.22, 151.60, 127.41, 116.04, 112.36. Elemental analysis for C_6_H_6_N_4_O_4_ (198.14); calculated: C, 36.37; H, 3.05; N, 28.28, found C, 36.41; H, 3.09; N, 28.25.

(*E*)-2-[(5-Nitrofuran-2-yl)methylene]hydrazine-1-carbothioamide **3e**. Yellow solid; yield 89%; mp >250 °C (229 °C decomp. [47]). IR (ATR): 3462, 3300, 3086, 2977, 1602, 1592, 1570, 1537, 1504, 1481, 1451, 1393, 1352, 1273, 1257, 1189, 1104, 1058, 1028, 972, 909, 845, 833, 812, 777, 733, 652 cm^−1^. ^1^H NMR (600 MHz, DMSO-*d*_6_): δ 11.80 (1H, s, CSNHN), 8.48 (1H, s, CS-NH_A_), 7.99 (1H, s, CS-NH_B_), 7.93 (1H, s, CH = N), 7.75 (1H, d, *J* = 3.9 Hz, H4), 7.33 (1H, d, *J* = 4.0 Hz, H3). ^13^C NMR (151 MHz, DMSO): δ 178.95, 153.15, 152.11, 130.33, 115.67, 113.74. Elemental analysis for C_6_H_6_N_4_O_3_S (214.20); calculated: C, 33.64; H, 2.82; N, 26.16, found C, 33.61; H, 2.85; N, 26.16.

### 3.2. Antimicrobial Activity

#### 3.2.1. Antibacterial Activity

Antibacterial activity of **1**–**3** as well as five parent hydrazine compounds was evaluated against four Gram-positive and four Gram-negative bacterial strains of a clinical importance, namely: methicillin-susceptible *Staphylococcus aureus* subsp. *aureus* ATCC (American Type Culture Collection) 29213, CCM (Czech Collection of Microorganisms) 4223 (MSSA), methicillin-resistant *Staphylococcus aureus* subsp. *aureus* (MRSA) ATCC 43300, CCM 4750, *Staphylococcus epidermidis* ATCC 12228, CCM 4418, *Enterococcus faecalis* ATCC 29212, CCM 4224; *Escherichia coli* ATCC 25922, CCM 3954, *Klebsiella pneumoniae* ATCC 10031, CCM 4415, *Acinetobacter baumannii* ATCC 19606, DSM (German Collection of Microorganisms and Cell Cultures) 30007, and *Pseudomonas aeruginosa* ATCC 27853, CCM 3955. The strains were obtained from the CCM (Brno, Czech Republic) or DSM (Braunschweig, Germany).

The microdilution broth method was performed according to EUCAST (The European Committee on Antimicrobial Susceptibility Testing) instructions [48] with minor modifications at the concentration range of investigated compounds being 0.49–500 µM depending on their solubility; for several derivatives, the highest possible concentration was 125 µM. Briefly, the cultivation was done in Cation-adjusted Mueller-Hinton broth (CAMHB, M-H 2 Broth, Sigma-Aldrich, St. Louis, MO, USA) at 35 ± 2 °C. Tested compounds were dissolved in DMSO (Sigma-Aldrich, St. Louis, MO, USA) to produce stock solutions. The final concentration of DMSO in the testing medium did not exceed 1% (*v*/*v*) of the total solution composition and did not affect the growth of bacteria. Positive (microbes solely), negative (cultivation medium and DMSO) controls and internal quality standards were involved in each assay. Antibacterial activity is expressed as minimum inhibitory concentration (MIC, reported in µM) after 24 and 48 h of static incubation in dark and humidified atmosphere at 35 ± 2 °C. The experiments were performed in duplicates. For the results to be valid, the difference in MIC determined from two parallel measurements must not be greater than one step on the dilution scale. MIC was determined by naked eye in the well with the lowest drug concentration, where no visible growth of microbial agent was detected.

Broad-spectrum beta-lactam antibiotic piperacillin (PIP) was employed as a reference compound. The results of PIP were read after 24 h. Standard antibiotics (ciprofloxacin and gentamicin; data not shown) and internal quality control/reference strains are routinely included in parallel testing-basic screening of antibacterial activity.

#### 3.2.2. Antifungal Activity

Antifungal activity was evaluated against four yeasts, namely: *Candida albicans* ATCC 24443, CCM 8320, *Candida krusei* ATCC 6258, CCM 8271, *Candida parapsilosis* ATCC 22019, CCM 8260, *Candida tropicalis* ATCC 750, CCM 8264; and four strains of filamentous fungi, namely: *Aspergillus fumigatus* ATCC 204305, *Aspergillus flavus* CCM 8363, *Lichtheimia corymbifera* CCM 8077, and *Trichophyton interdigitale* ATCC 9533, CCM 8377. A microdilution broth method was performed according to EUCAST instructions [49,50] with minor modification. Briefly, tested compounds were dissolved in DMSO and diluted in a twofold manner with RPMI-1640 medium with l-glutamine, supplemented with 2% glucose (w/v) and buffered to pH 7.0 with 3-(*N*-morpholino)propane-1-sulfonic acid (all components were purchased from Sigma-Aldrich, St. Louis, MO, USA). The final concentration of DMSO in the tested medium did not exceed 1% (*v*/*v*) of the total solution composition, and it was confirmed that this concentration did not inhibit the fungal growth. Static incubation was performed in dark and humidified atmosphere, at 35 ± 2 °C for 24 and 48 h (72 and 120 h for *Trichophyton interdigitale*). Positive controls consisted of test fungus solely, while negative controls consisted of medium and DMSO. Internal quality control was included too. Standard antimycotic drugs (amphotericin B and voriconazole) such as internal quality controls were included in assays, too (data not shown). Visual inspection was used for MIC endpoints evaluation. The experiments were conducted in duplicates. For the results to be valid, the difference in MIC determined from two parallel measurements must not be greater than one step on the dilution scale. MIC was determined by naked eye in the well with the lowest drug concentration, where no visible growth of microbial agent was detected. MIC determination scale was identical as for antibacterial activity.

Parent five hydrazine derivatives and antifungal triazole drug fluconazole were involved as reference compounds. Antifungal activities of fluconazole are presented as mean 50% growth inhibition. Results were read after 24 h (yeasts) or 48 h (moulds) microdilution plates cultivation without agitation at 35 ± 2 °C in humidified atmosphere. The results were read with a microdilution plate reader (SynergyTM HTX, BioTek Instruments, Inc., VT, USA) at wavelength 530 nm.

### 3.3. Evaluation of Acetyl- and Butyrylcholinesterase Inhibition

The activities of the compounds **1**–**3** were measured via the Ellman’s spectrophotometric method that was modified according to Zdražilová et al. [51]. The activity of cholinesterase enzymes is assessed indirectly by quantifying the amount of 5-mercapto-2-nitrobenzoic acid ion formed in the reaction of the thiol reagent 5,5′-disulfanediylbis(2-nitrobenzoic acid) (DTNB) with thiocholine, a product of the hydrolysis of acetylthiocholine (ATCh) or butyrylthiocholine (BTCh) catalysed by AChE and BuChE, respectively.

AChE was obtained from electric eel (*Electrophorus electricus* L.) and BuChE from equine serum. The benzazepine alkaloid galantamine and carbamate drug rivastigmine were used as reference enzyme inhibitors. These compounds are approved for treatment of Alzheimer’s disease. All the enzymes, the thiol reagent, ATCh, BTCh, rivastigmine and galantamine were purchased from Sigma-Aldrich.

The enzyme activity in final reaction mixture (2 mL) was 0.2 U/mL, the concentration of ATCh or BTCh 40 µM and the concentration of DTNB 0.1 mM for all reactions. The investigated hydrazide-hydrazones were dissolved in DMSO and then diluted by demineralized water (conductivity 3 μS) to the concentration of 1 mM. For all of them as well as galantamine and rivastigmine five different concentrations of inhibitor in final reaction mixture were used. Final DMSO concentration in the reaction mixture was 0.3%. All measurements were carried in triplicates and the average values of reaction rate (v_0_-uninhibited reaction, v_i_-inhibited reaction) were used for construction of the dependence v_0_/v_i_ vs. concentration of inhibitor. From obtained equation of regression curve, IC_50_ values were calculated.

For the determination of the type of inhibition of the selected inhibitors (**1n**, **1m**, **1p**, **2a**, and **2e**), Lineweaver–Burk plot was used, and the measuring procedure was analogous to the determination of IC_50_. The enzyme activity in the final reaction mixture (2 mL) was 0.2 U/mL, a concentration of ATCh and BTCh 20–80 µM and a concentration of DTNB was 0.1 mM. For each of the substrate concentrations, four different concentrations of the inhibitor were used (0.5, 1, 1.5, and 2 µM for AChE, and predominantly 1, 2, 3 and 4 µM for BuChE). The dependence absorbance vs. time was observed and the reaction rate was calculated. All measurements were carried in duplicates and the average values of reaction rate were used for the construction of the Lineweaver–Burk plot. From obtained equations of regression curves, the values of Michaelis constant (K_M_) and maximum velocity (V_m_) were calculated, and the type of inhibition was identified.

### 3.4. Molecular Docking

Crystallographic structures of human AChE and human BChE were obtained from protein data bank (www.rcsb.org, accessed on 26 January 2021; pdb codes 4PQE and 1POI, respectively). The 3D structures of ligands were prepared in Chem3D Pro 19.1 (ChemBioOffice 2019 Package, CambridgeSoft, Cambridge, MA, USA). In the preparation process all water molecules were removed from the enzymes and structures of enzymes and ligands were optimized using UCSF Chimera software package (Amber force field) [52]. Docking was performed using Autodock Vina [53] and Autodock 4.2 [54] (a Lamarckian genetic algorithm was used). The 3D affinity grid box was designed to include the full active and peripheral site of AChE and BChE. The number of grid points in the x-, y- and z-axes was 20, 20 and 20 with grid points separated by 1 Å (Autodock Vina) and 40, 40 and 40 with grid points separated by 0.4 Å (Autodock 4). The graphic visualisations of the ligand-enzyme interactions were prepared in PyMOL (The PyMOL Molecular Graphics System, Version 1.5 Schrödinger, LLC., New York, NY, USA).

### 3.5. Cytotoxicity Determination

The human hepatocellular liver carcinoma cell line HepG2 (passage 6–8 for HepG2 cells, 20–23 for LDH assay) purchased from Health Protection Agency Culture Collections (ECACC, Salisbury, UK) was cultured in minimum essentials eagle medium (MEM) supplemented with 10% fetal bovine serum, 1% L-glutamine solution and non-essential amino acid solution in a humidified atmosphere containing 5% CO_2_ at 37 °C. For subculturing, the cells were harvested after trypsin/EDTA treatment at 37 °C. All used chemicals were obtained from Sigma-Aldrich (St. Louis, MO, USA). To evaluate cytotoxicity of the compounds, the cells treated with the tested substances were used as experimental groups whereas untreated cells served as control groups. The range of concentrations tested were 1–500 µM (**1m**, **1o**, **1p**, **2c**, **2e**, **3e**, and tamoxifen) or 1–250 µM for **1n** and **2a**.

The cells were seeded in a density of 15,000 cells per well in a 96-well plate. The next day the cells were treated with each of the tested substances at a broad range of concentrations (based on their solubility) in triplicates. The compounds were dissolved in DMSO (maximum incubation concentration of DMSO was 1% *v*/*v*). The controls representing 100% cell viability, 0% cell viability (treated with 10% DMSO), no cell control and vehiculum control were incubated in parallel also in triplicates. After 24 h of incubation in a humidified atmosphere containing 5% CO_2_ at 37 °C, the reagent from the kit CellTiter 96 AQueous One Solution Cell Proliferation Assay^®^ (CellTiter 96; PROMEGA, Fitchburg, WI, USA) was added. After 2 h of incubation at 37 °C, absorbance of samples was recorded at 490 nm (TECAN, Infinita M200, Grödig, Austria). A standard toxicological parameter IC_50_ was calculated by nonlinear regression from a semilogarithmic plot of incubation concentration versus percentage of viability relative to untreated controls using GraphPad Prism 8 software (GraphPad Software, Inc., La Jolla, CA, USA).

Results of the experiments are presented as inhibitory concentrations that reduce viability of the cell population to 50% from the maximal viability (IC_50_). Known cytotoxic drug tamoxifen (as citrate salt) was involved as a reference compound.

For evaluating effect on membrane integrity (LDH release), the CytoTox 96^®^ Non-Radioactive Cytotoxicity Assay (PROMEGA, Fitchburg, WI, USA) was performed. The range of concentrations tested was 1–500 µM. After 24 h of incubation at 37 °C, 50 μL of medium was removed and 50 μL of reagent was added, and then after 30 min the reaction was stopped and the absorbance of the samples at 490 nm was recorded (TECAN). The standard toxicological parameter EC_50_ was calculated by non-linear regression from a semi-logarithmic plot of incubation concentration versus percent absorbance relative to untreated controls using GraphPad Prism 9 software (GraphPad Software, Inc., La Jolla, CA, USA).

## 4. Conclusions

Based on the previous reports of especially robenidine analogues [9,22] and our experience with halogenated salicylidene derivatives, we designed and synthesized their analogues derived from chlorobenzaldehydes, halogenated salicylaldehydes, 5-nitrofurfural, isatin, plus related aldehydes and AG, 1,3-diaminoguanidine, nitroaminoguanidine, and (thio)semicarbazone as potential bioactive compounds. In summary, we have prepared thirty-one compounds, in most cases hydrazones, fifteen of which have not yet been reported previously. Some of the known derivatives have not yet been biologically evaluated.

Initially, we were focused on antimicrobial evaluation against Gram-positive and Gram-negative bacteria, yeasts, and filamentous fungi. The compounds showed broad-spectrum of antibacterial activity with MIC from 7.81 µM; Gram-positive strains including MRSA were more susceptible. The best activity was associated with 3,5-diiodobenzylidene, 5-nitrofurylidene, and (5-nitrothiophen-2-yl)methylidene scaffolds. However, results were beyond our expectations based on previous reports. Antifungal activity was rather mild with MIC from 62.5 µM; 3,5-diiodosalicylidene scaffold was the optimal choice.

Then, we evaluated inhibition of AChE and BuChE. The compounds have been identified as potent to moderate inhibitors of both AChE and BuChE with a variable selectivity depending on substitution pattern. 5-Nitrofurylidene led to selective inhibition of AChE, while *N*’-(2-hydroxy-3,5-diiodobenzylidene)hydrazinecarboximidhydrazide was the most potent and also selective BuChE inhibitor. Isatin-AG condensate was the most active against AChE along with identical IC_50_ for of BuChE. Having these results in hand, we evaluated type of inhibition of the most active compounds with the conclusion that they are mixed-type inhibitors. Molecular docking provided insight into their interactions with the enzymes. The hydrazones bind closely to intrinsic anionic site and PAS of AChE, while in BuChE they are bound near the entrance to the active gorge.

Finally, the most promising hydrazones, along with their synthetic precursors, were screened for their toxicity in eukaryotic cell line. The compounds exhibited variable toxicity with IC_50_ ranging from low micromolar concentration (lower than obtained for tamoxifen) to non-toxic derivatives.

Taken together, several derivatives can be considered as promising hits especially for inhibition of cholinesterases and antibacterial properties. The coincidence of antimicrobial and cytotoxic actions of some compounds may also be perspective. The biological profile is clearly dependent on both parent amino and carbonyl compounds; it is possible to achieve target activity and to separate them. However, prior to extensive hit-to-lead optimization campaign, a caution of addressing potential membrane-damaging effect indicated for some compounds by LDH release assay and pan-assay interference (PAINS)-like behavior derived from several structural motifs should be excluded.

## Figures and Tables

**Table 1:** Antibacterial activity of **1**–**3**.

Code	MIC [µM]															
	SA		MRSA		SE		EF		EC		KP		ACI		PA	
	24 h	48 h	24 h	48 h	24 h	48 h	24 h	48 h	24 h	48 h	24 h	48 h	24 h	48 h	24 h	48 h
**1a**	500	500	>500	>500	250	250	>500	>500	>500	>500	>500	>500	>500	>500	>500	>500
**1f**	500	500	500	500	125	125	500	500	500	500	500	500	500	500	**500**	**500**
**1g**	250	250	500	500	62.5	62.5	**125**	250	500	500	500	500	500	500	**500**	**500**
**1h**	250	500	>500	>500	125	250	>500	>500	>500	>500	>500	>500	500	500	>500	>500
**1i**	125	125	125	125	250	250	500	500	250	250	250	250	500	500	**500**	**500**
**1j**	250	250	250	250	250	250	250	250	500	500	500	500	500	500	**250**	**250**
**1k**	250	250	250	250	125	250	500	500	500	500	250	250	>500	>500	>500	>500
**1l**	500	500	250	250	250	250	250	250	250	250	250	250	500	500	>500	>500
**1n**	**62.5**	125	125	125	62.5	125	500	500	**62.5**	**125**	250	250	**250**	500	**500**	**500**
**1o**	125	250	125	250	62.5	62.5	250	500	**62.5**	**62.5**	**125**	**125**	**125**	**125**	**500**	**500**
**2a**	125	125	125	125	250	250	250	250	250	250	250	250	500	500	>500	>500
**2b**	500	500	500	500	250	500	500	500	500	500	250	250	>500	>500	>500	>500
**2c**	**15.62**	**15.62**	**15.62**	**15.62**	**7.8**	**7.8**	**31.25**	**31.25**	**62.5**	**62.5**	**62.5**	**62.5**	**250**	**250**	**250**	**250**
**2e**	**62.5**	125	125	125	**31.25**	**31.25**	>125	>125	>125	>125	>125	>125	>125	>125	>125	>125
**2h**	250	250	500	500	125	125	>500	>500	>500	>500	>500	>500	>500	>500	>500	>500
**2i**	500	500	500	500	250	250	250	250	>500	>500	>500	>500	>500	>500	>500	>500
**3d**	**62.5**	125	**62.5**	125	**31.25**	62.5	500	500	**125**	250	**62.5**	**125**	>500	>500	>500	>500
**3e**	**62.5**	**62.5**	**62.5**	**62.5**	**31.25**	62.5	**125**	**125**	**125**	250	**62.5**	**62.5**	>500	>500	>500	>500
**PIP**	1.85		14.83		>59.31		1.85		7.41		>59.31		>59.31		14.83	

PIP = piperacillin sodium salt; SA: *Staphylococcus aureus* ATCC 29213; MRSA: methicillin-resistant *Staphylococcus aureus* ATCC 43300; SE: *Staphylococcus epidermidis* ATCC 12228; EF: *Enterococcus faecalis* ATCC 29212; EC: *Escherichia coli* ATCC 25922; KP: *Klebsiella pneumoniae* ATCC 10031; ACI: *Acinetobacter baumannii* ATCC 19606; PA: *Pseudomonas aeruginosa* ATCC 37853. The lowest MIC values for each strain are given in bold.

**Table 2:** Antifungal activity of **1**–**3**.

Code	MIC [µM]									
	CA		CK		CP		CT		TI	
	24 h	48 h	24 h	48 h	24 h	48 h	24 h	48 h	72 h	120 h
**1i**	500	500	**250**	**250**	**250**	**250**	500	500	**62.5**	**125**
**1k**	**250**	500	500	500	500	500	500	500	**125**	250
**1l**	>500	>500	500	500	500	500	500	500	**125**	**125**
**1o**	>500	>500	500	500	>500	>500	>500	>500	250	250
**2a**	**125**	**125**	500	500	500	500	500	500	**62.5**	**125**
**2d**	>125	>125	>125	>125	>125	>125	>125	>125	**62.5**	**62.5**
**2e**	**125**	**125**	**125**	**125**	**125**	**125**	**125**	**125**	**125**	**125**
**2g**	>500	>500	>500	>500	>500	>500	>500	>500	250	250
FLU *	6.5	6.5	>104.5	>104.5	3.3	3.3	6.5	6.5	52.2	52.2

FLU = fluconazole. *: values correspond to IC_50_ (50% growth inhibition). CA: *Candida albicans* ATCC 24433; CK: *Candida krusei* ATCC 6258; CP: *Candida parapsilosis* ATCC 22019; CT: *Candida tropicalis* ATCC 750; TI: *Trichophyton interdigitale* ATCC 9533. One or two of the best MIC value(s) for each strain are shown in bold.

**Table 3:** Inhibition of AChE and BuChE and selectivity indexes of **1**–**3**.

Code	IC_50_ AChE (µM)	IC_50_ BuChE (µM)	Selectivity AChE/BuChE
**1a**	36.19 ± 0.10	44.95 ± 0.54	0.81
**1b**	49.35 ± 0.11	99.12 ± 3.63	0.50
**1c**	43.89 ± 0.76	41.47 ± 3.06	1.06
**1d**	45.20 ± 1.60	13.89 ± 0.28	3.25
**1e**	46.23 ± 2.26	**5.57 ± 0.24**	**8.30**
**1f**	42.79 ± 1.33	30.61 ± 1.51	1.40
**1g**	37.43 ± 0.73	45.22 ± 0.86	0.83
**1h**	29.49 ± 0.93	62.28 ± 1.08	0.47
**1i**	**25.03 ± 0.50**	19.76 ± 0.31	1.27
**1j**	40.84 ± 0.80	61.74 ± 4.33	0.66
**1k**	**22.43 ± 0.67**	11.68 ± 0.16	1.92
**1l**	**22.13 ± 0.55**	**5.42 ± 0.17**	4.08
**1m**	**29.05 ± 2.49**	**3.57 ± 0.03**	**8.14**
**1n**	**24.15 ± 0.75**	203.99 ± 13.20	0.12
**1o**	31.67 ± 1.34	286.05 ± 16.41	0.11
**1p**	**17.95 ± 0.90**	17.51 ± 0.21	1.03
**2a**	**20.53 ± 0.48**	**4.52 ± 0.27**	4.54
**2b**	40.30 ± 0.85	46.28 ± 0.29	0.87
**2c**	**29.03 ± 1.19**	85.54 ± 1.46	0.34
**2d**	45.67 ± 1.70	41.52 ± 1.45	1.10
**2e**	**28.16 ± 0.98**	**1.69 ± 0.17**	**16.66**
**2f**	34.78 ± 0.04	>500	**<0.07**
**2g**	54.93 ± 1.72	**8.26 ± 0.21**	6.65
**2h**	30.72 ± 0.33	34.72 ± 0.35	0.88
**2i**	52.22 ± 0.40	86.24 ± 0.39	0.61
**3a**	41.08 ± 0.54	82.30 ± 3.02	0.50
**3b**	**24.75 ± 0.17**	>500	**<0.05**
**3c**	39.98 ± 2.56	>500	**<0.08**
**3d**	36.60 ± 0.69	>500	**<0.07**
**3e**	33.16 ± 0.06	>500	**<0.07**
aminoguanidine	75.14 ± 0.74	326.55 ± 18.02	0.23
1,3-diaminoguanidine	41.71 ± 0.23	283.32 ± 8.94	0.15
nitroaminoguanidine	46.87 ± 0.44	>500	**<0.09**
semicarbazide	48.08 ± 0.84	364.63 ± 16.82	0.13
thiosemicarbazide	46.11 ± 1.10	327.78 ± 3.22	0.14
rivastigmine	56.10 ± 1.41	38.40 ± 1.97	1.46
galantamine	1.54 ± 0.02	2.77 ± 0.15	0.56

IC_50_ values are expressed as the mean ± SD (*n* = three independent experiments). The lowest IC_50_ values for each enzyme are given in bold as well as the most selective inhibitors for both enzymes.

**Table 4:** Binding energies of selected ligands to AChE and BuChE.

Code	Affinity for AChE (kcal.mol^−1^)	Affinity for BuChE (kcal.mol^−1^)
**1m**	−7.7	−8.1
**1p**	−9.1	−7.6
**2a**	−8.5	−8.2
**2e**	−8.0	−8.3
**3b**	−8.0	−7.2

**Table 5:** Cytotoxicity of the selected derivatives **1**–**3** towards HepG2 cell line.

Code	IC_50_ [µM]	Code	IC_50_ [µM]
**1m**	56.52	**2c**	9.84
**1n**	10.41	**2e**	119.6
**1o**	5.27	**3e**	29.78
**1p**	˃500	**tamoxifen**	19.6
**2a**	31.29		

**Table 6:** Cytotoxicity of the selected derivatives **1**–**3** line using LDH release assay.

Code	EC_50_ [µM]	Code	EC_50_ [µM]
**1m**	21.26	**2a**	30.43
**1n**	3.55	**2c**	5.44
**1o**	0.79	**2e**	131.9
**1p**	129.5	**3e**	5.25

## Data Availability

Data is contained within the article or Supplementary Materials.

## Supplementary Materials

The following are available online at https://www.mdpi.com/article/10.3390/ph14121229/s1, Figure S1. ^1^H NMR spectrum (500 MHz, DMSO- *d*_6_) of (*E*)-2-(6-chloro-2-hydroxybenzylidene)hydrazine-1-carboximidamide **1h**, Figure S2. ^13^C NMR spectrum (126 MHz, DMSO- *d*_6_) of (*E*)-2-(6-chloro-2-hydroxybenzylidene)hydrazine-1-carboximidamide **1h**, Figure S3. ^1^H NMR spectrum (600 MHz, DMSO- *d*_6_) of (*E*)-2-[(5-nitrofuran-2-yl)methylene]hydrazine-1-carboximidamide **1n**. Figure S4. ^13^C NMR spectrum (151 MHz, DMSO- *d*_6_) of (*E*)-2-[(5-nitrofuran-2-yl)methylene]hydrazine-1-carboximidamide **1n**, Figure S5. ^1^H NMR spectrum (600 MHz, DMSO- *d*_6_) of (*E*)-2-(3,5-diiodobenzylidene)hydrazine-1-carboximidamide **2c**, Figure S6. ^13^C NMR spectrum (151 MHz, DMSO- *d*_6_) of (*E*)-2-(3,5-diiodobenzylidene)hydrazine-1-carboximidamide **2c**, Figure S7. ^1^H NMR spectrum (600 MHz, DMSO- *d*_6_) of (*E*)-2-(2-hydroxy-3,5-diiodobenzylidene)-*N*-nitrohydrazine-1-carboximidamide **2f**, Figure S8. ^13^C NMR spectrum (151 MHz, DMSO- *d*_6_) of (*E*)-2-(2-hydroxy-3,5-diiodobenzylidene)-*N*-nitrohydrazine-1-carboximidamide **2f**, Figure S9. ^1^H NMR spectrum (600 MHz, DMSO- *d*_6_) of (*E*)-2-(2-hydroxy-3,5-diiodobenzylidene)hydrazine-1-carboxamide **2h**, Figure S10. ^13^C NMR spectrum (151 MHz, DMSO- *d*_6_) of (*E*)-2-(2-hydroxy-3,5-diiodobenzylidene)hydrazine-1-carboxamide **2h**.
