# Supplementary material for: Novel Aminoguanidine Hydrazone Analogues: From Potential Antimicrobial Agents to Potent Cholinesterase Inhibitors
Source: Pharmaceuticals (Basel). 2021 Nov 26;14(12):1229. doi: 10.3390/ph14121229 (PMC8704707; doi:10.3390/ph14121229)

Novel Aminoguanidine Hydrazone Analogues: From Potential Antimicrobial Agents to Potent Cholinesterase Inhibitors

Martin Krátký [1,*], Šárka Štěpánková [2], Klára Konečná [3], Katarína Svrčková [2], Jana Maixnerová [4], Markéta Švarcová [1,5], Ondřej Janďourek [3], František Trejtnar [4] and Jarmila Vinšová [1]

[1] Department of Organic and Bioorganic Chemistry, Faculty of Pharmacy in Hradec Králové, Charles University, Akademika Heyrovského 1203, 500 05 Hradec Králové, Czech Republic; komloova@seznam.cz (M.Š.); vinsova@faf.cuni.cz (J.V.)

[2] Department of Biological and Biochemical Sciences, Faculty of Chemical Technology, University of Pardubice, Studentská 573, 532 10 Pardubice, Czech Republic; sarka.stepankova@upce.cz (Š.Š.); katarina.svrckova@upce.cz (K.S.)

[3] Department of Biological and Medical Sciences, Faculty of Pharmacy in Hradec Králové, Charles University, Akademika Heyrovského 1203, 500 05 Hradec Králové, Czech Republic; konecna@faf.cuni.cz (K.K.); jando6aa@faf.cuni.cz (O.J.)

[4] Department of Pharmacology and Toxicology, Faculty of Pharmacy in Hradec Králové, Charles University, Akademika Heyrovského 1203, 500 05 Hradec Králové, Czech Republic; maixj6a1@faf.cuni.cz (J.M.); trejtnarf@faf.cuni.cz (F.T.)

[5] Department of Chemistry, Faculty of Science, J. E. Purkinje University, Pasteurova 3632/15, 400 96 Ústí nad Labem, Czech Republic

1. NMR spectra of the representative compounds

Figure. S1. ^{1}H NMR spectrum (500 MHz, DMSO-d_6) of (*E*)-2-(6-chloro-2-hydroxybenzylidene)hydrazine-1-carboximidamide **1h**.

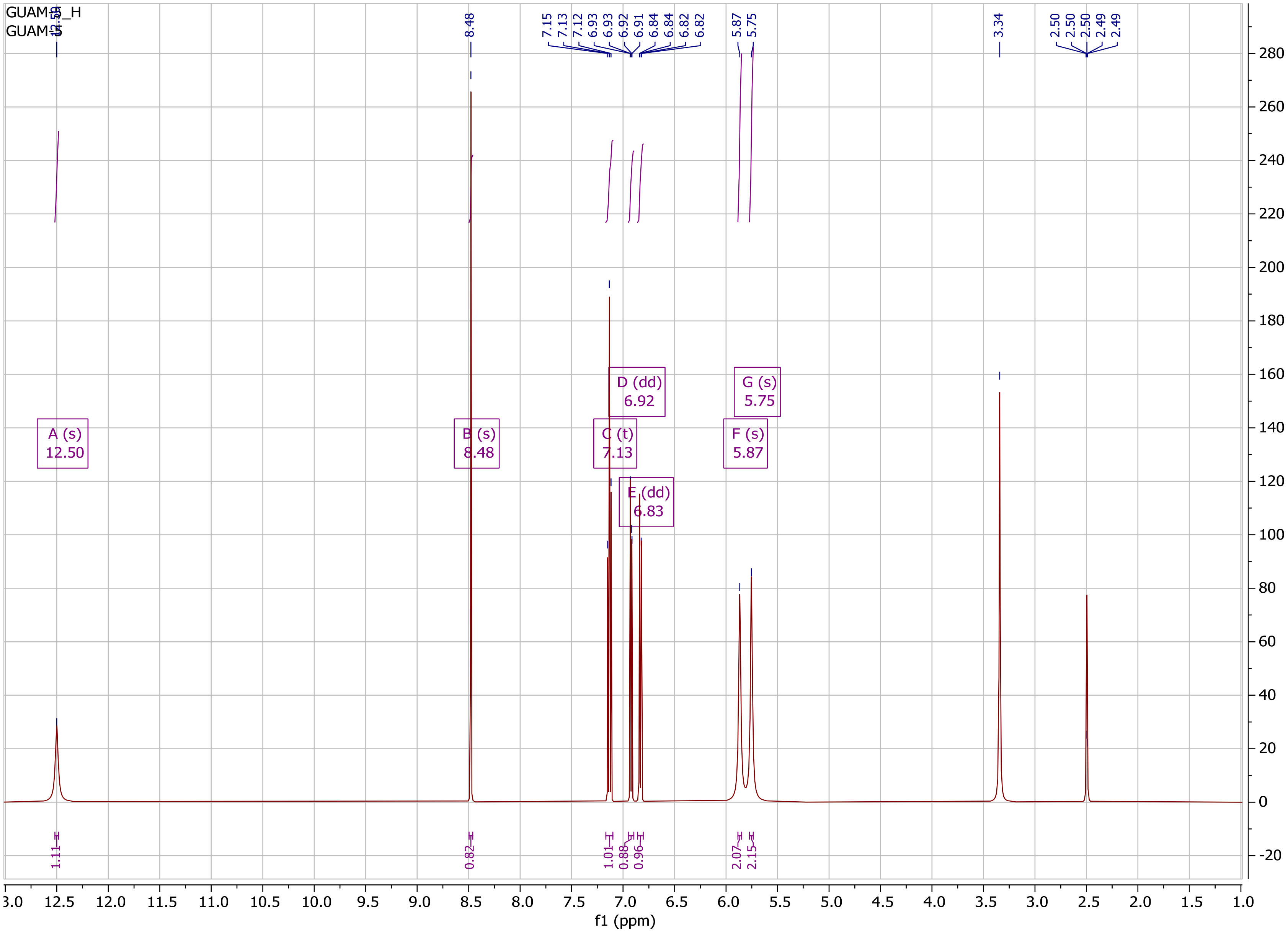

Figure. S2. ^{13}C NMR spectrum (126 MHz, DMSO-*d*$_6$) of (*E*)-2-(6-chloro-2-hydroxybenzylidene)hydrazine-1-carboximidamide **1h**.

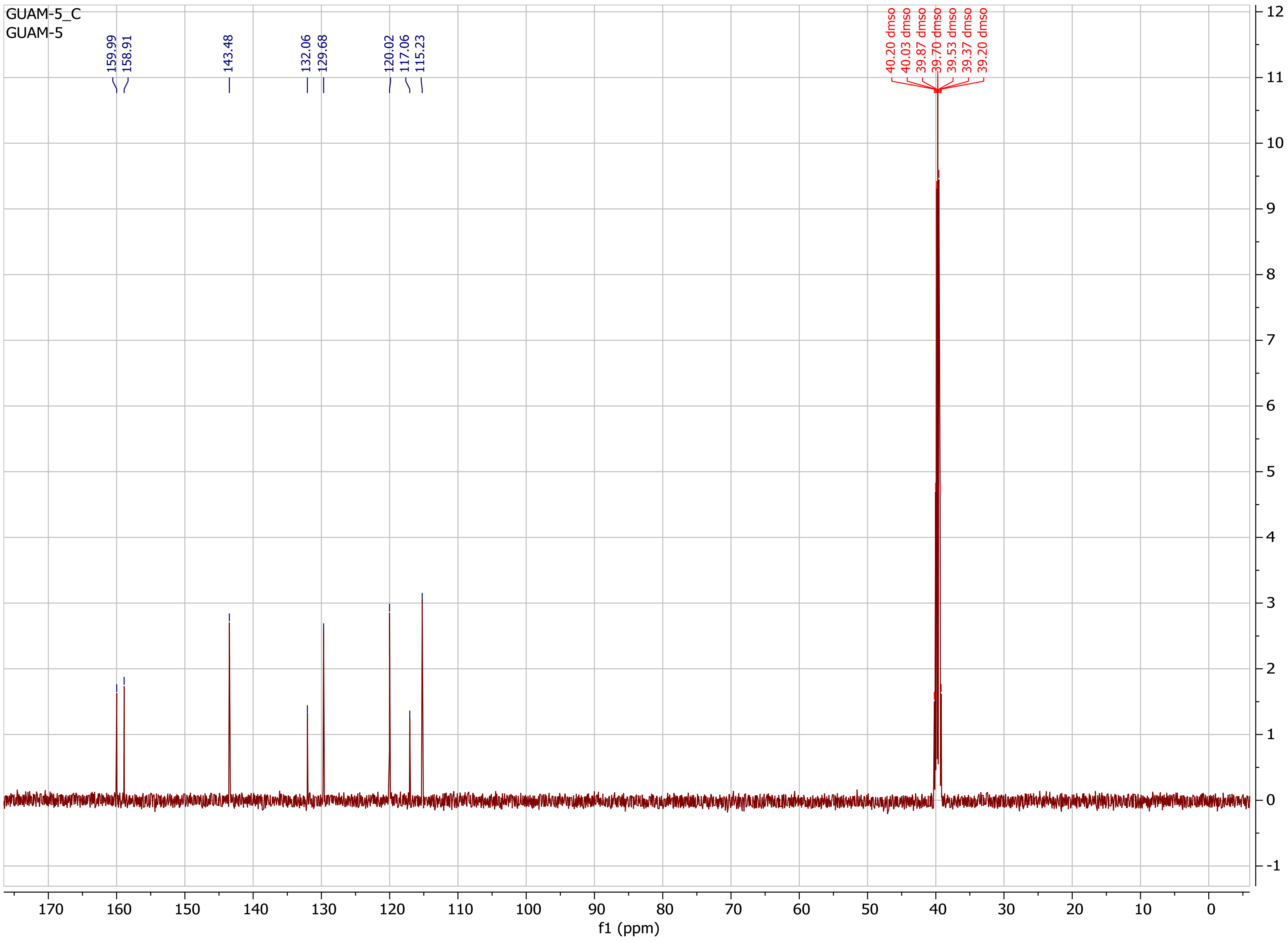

Figure. S3. ^{1}H NMR spectrum (600 MHz, DMSO-d_6) of (*E*)-2-[(5-nitrofuran-2-yl)methylene]hydrazine-1-carboximidamide **1n**.

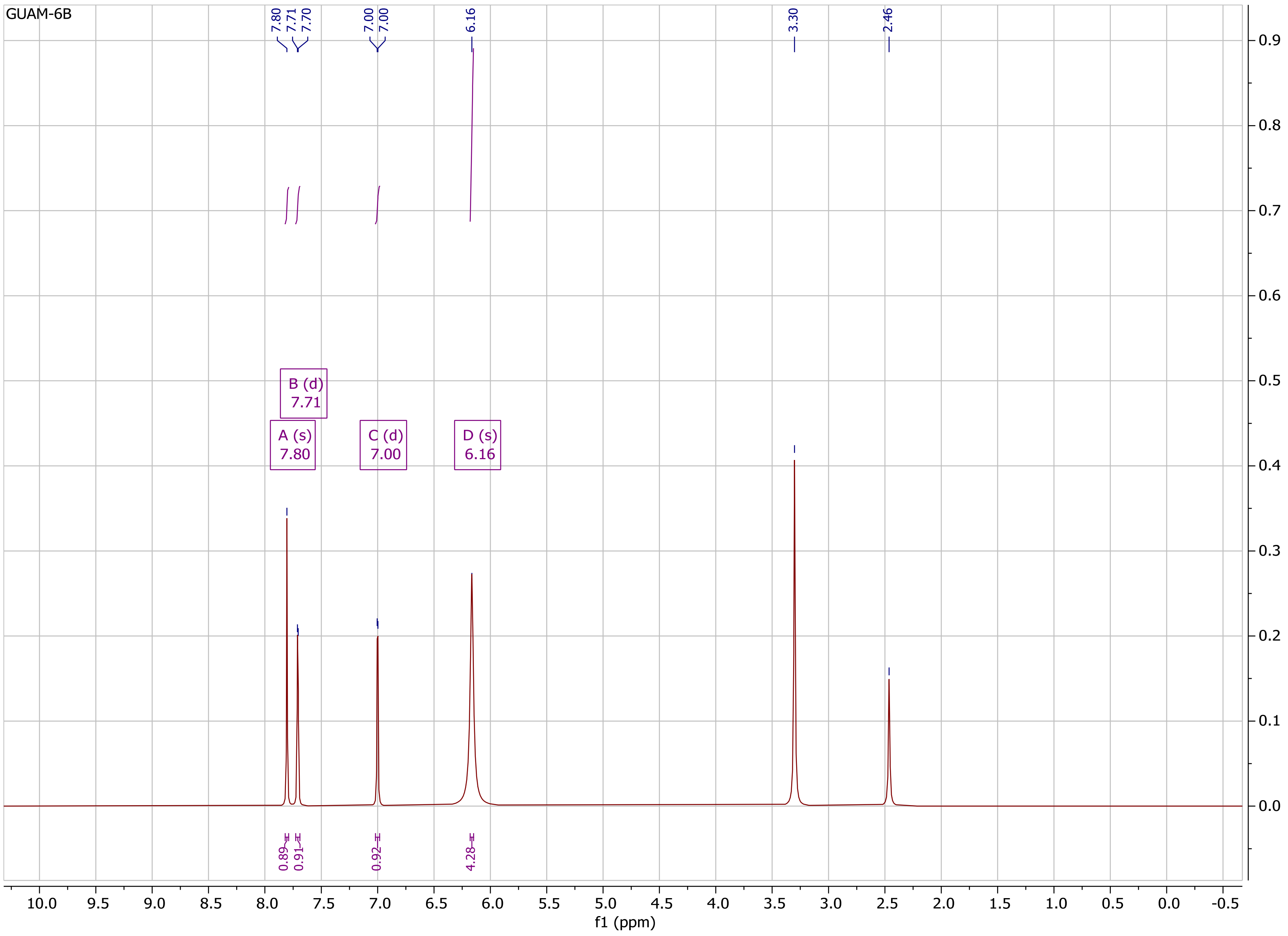

Figure. S4. ^{13}C NMR spectrum (151 MHz, DMSO-d_6) of (*E*)-2-[(5-nitrofuran-2-yl)methylene]hydrazine-1-carboximidamide **1n**.

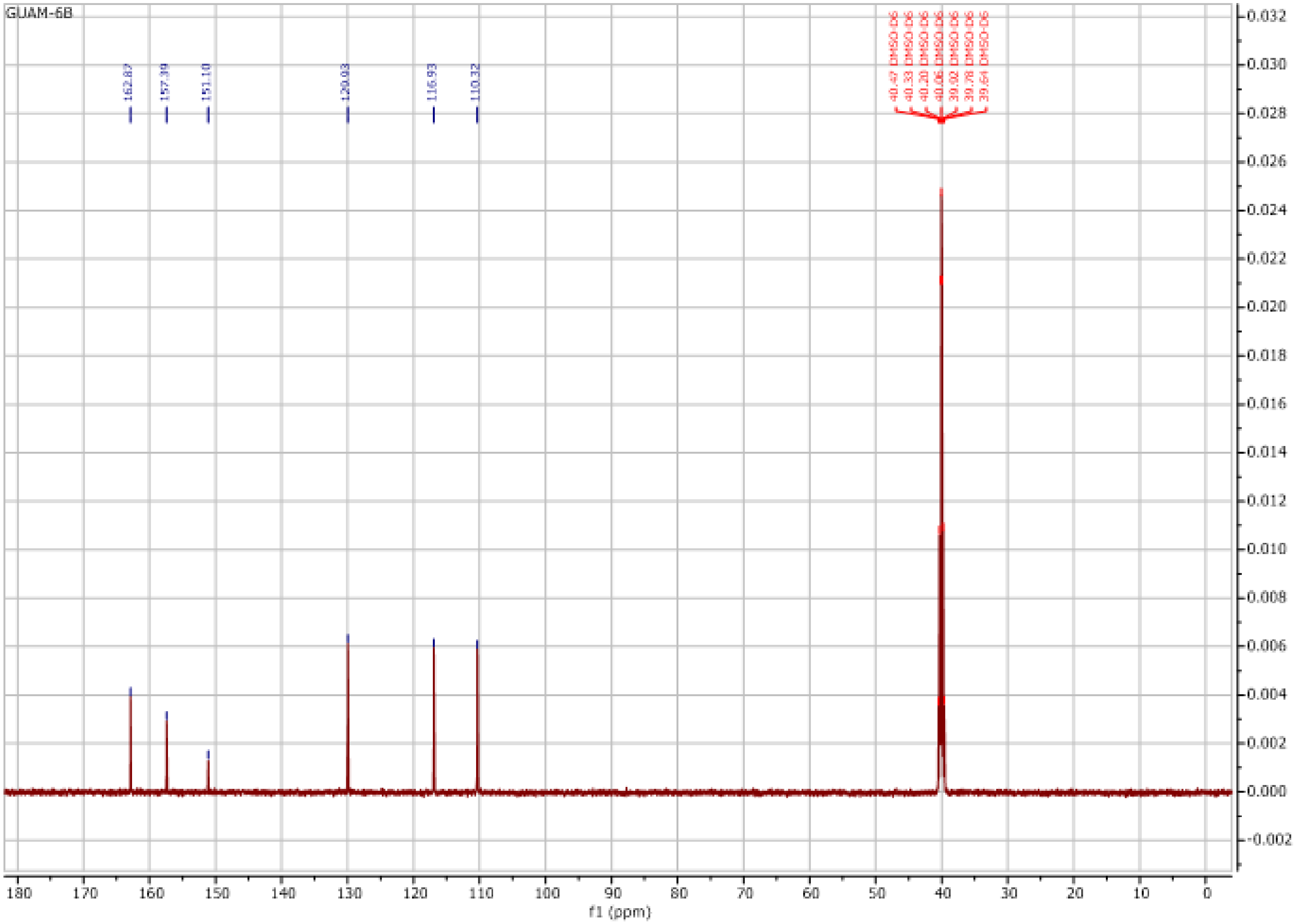

Figure. S5. ^{1}H NMR spectrum (600 MHz, DMSO-d_6) of (*E*)-2-(3,5-diiodobenzylidene)hydrazine-1-carboximidamide **2c**.

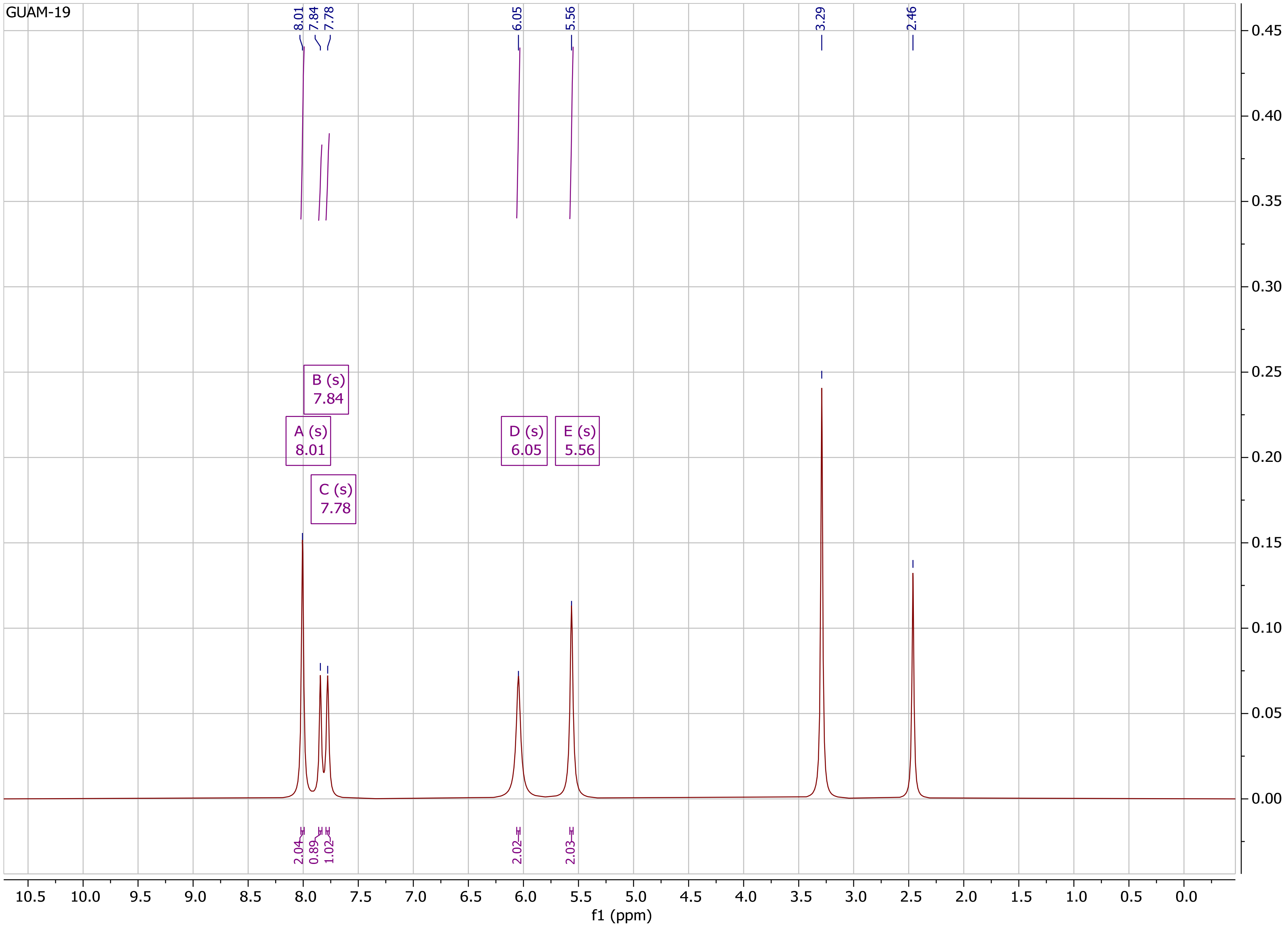

Figure. S6. ^{13}C NMR spectrum (151 MHz, DMSO-d_6) of (*E*)-2-(3,5-diiodobenzylidene)hydrazine-1-carboximidamide **2c**.

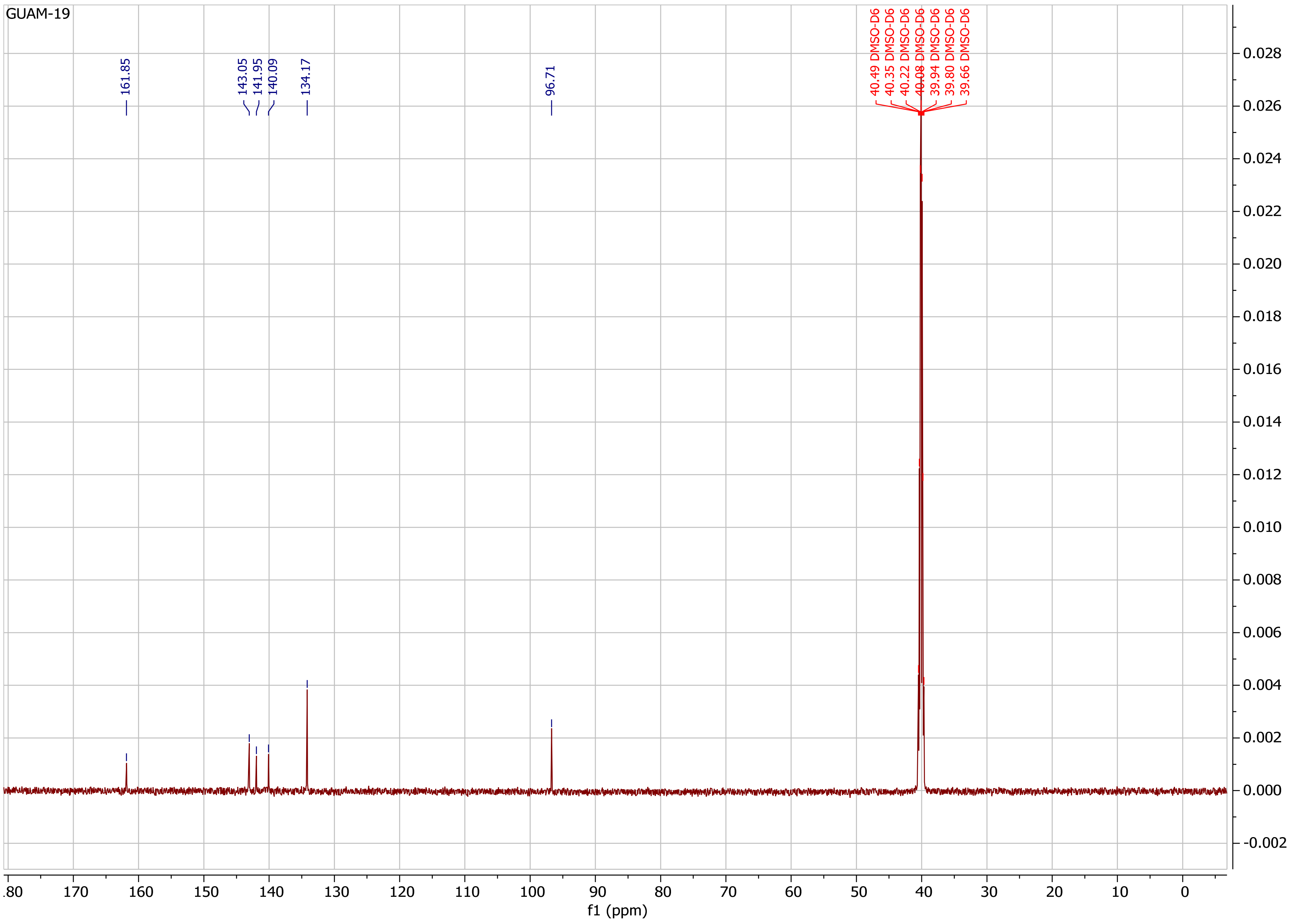

Figure. S7. ^{1}H NMR spectrum (600 MHz, DMSO-d_6) of (*E*)-2-(2-hydroxy-3,5-diiodobenzylidene)-*N*-nitrohydrazine-1-carboximidamide **2f**.

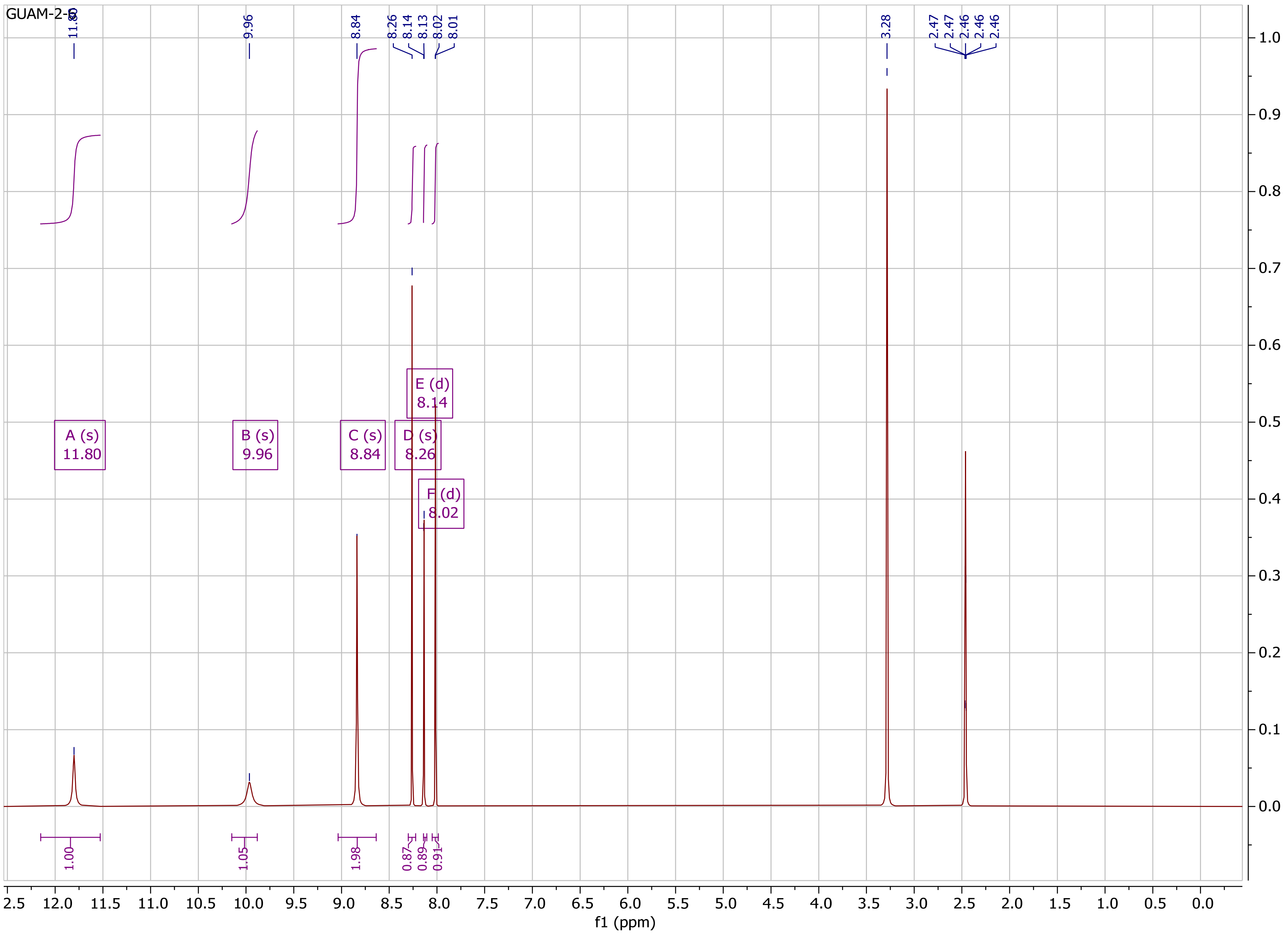

Figure. S8. ^{13}C NMR spectrum (151 MHz, DMSO-d_6) of (*E*)-2-(2-hydroxy-3,5-diiodobenzylidene)-*N*-nitrohydrazine-1-carboximidamide **2f**.

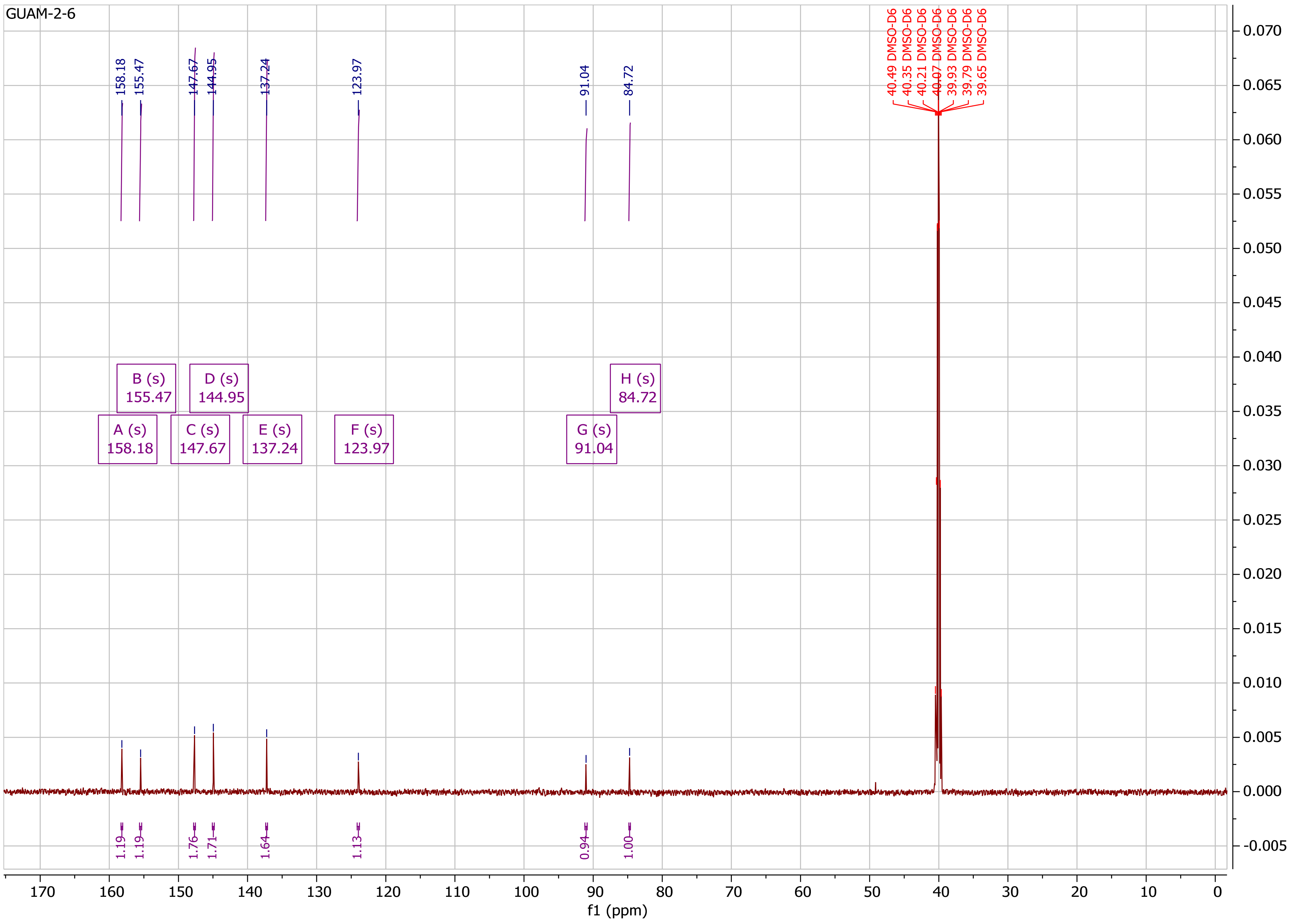

Figure. S9. ^{1}H NMR spectrum (600 MHz, DMSO-d_6) of (*E*)-2-(2-hydroxy-3,5-diiodobenzylidene)hydrazine-1-carboxamide **2h**.

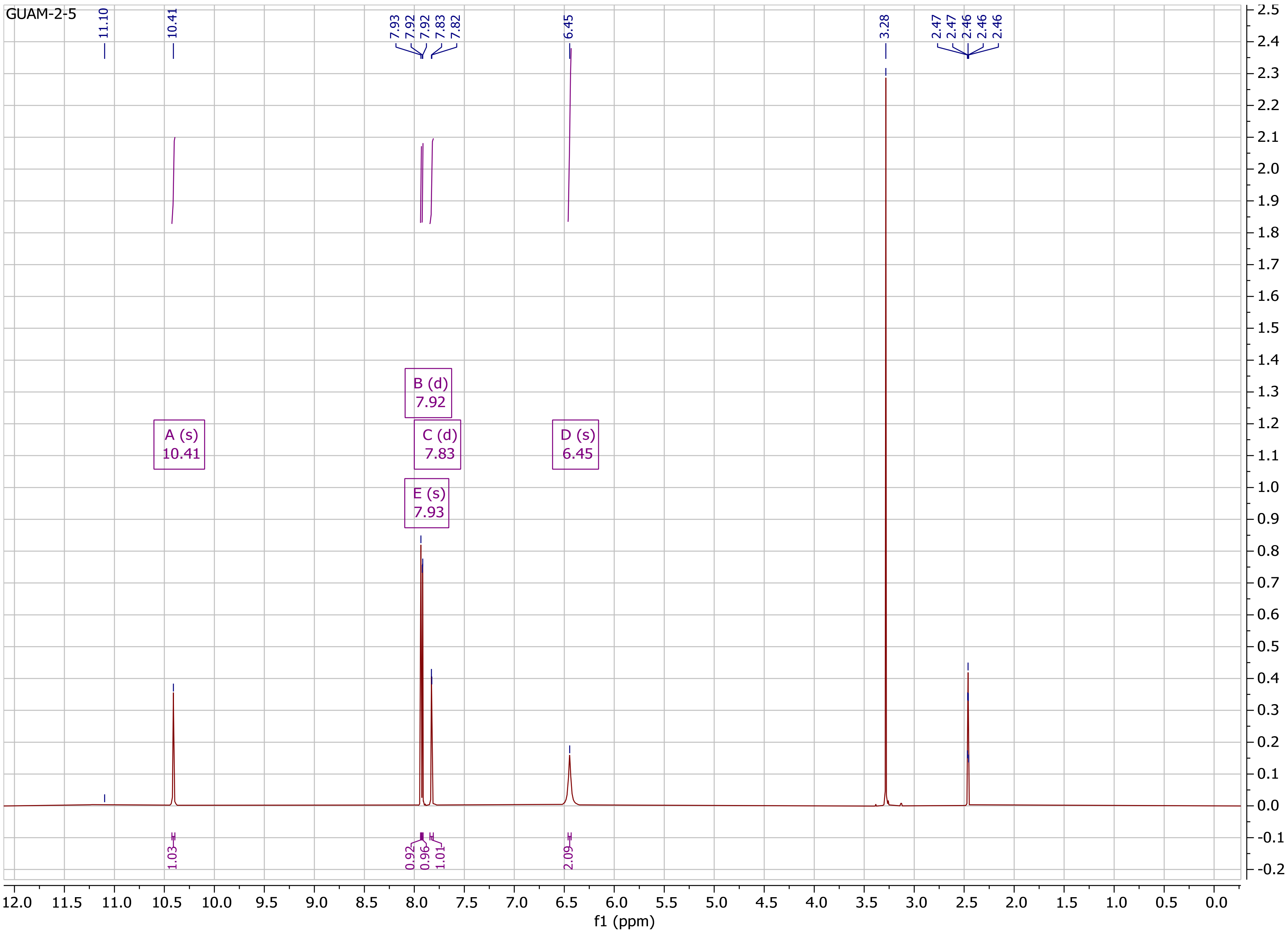

Figure. S10. ^{13}C NMR spectrum (151 MHz, DMSO-d_6) of (*E*)-2-(2-hydroxy-3,5-diiodobenzylidene)hydrazine-1-carboxamide **2h**.

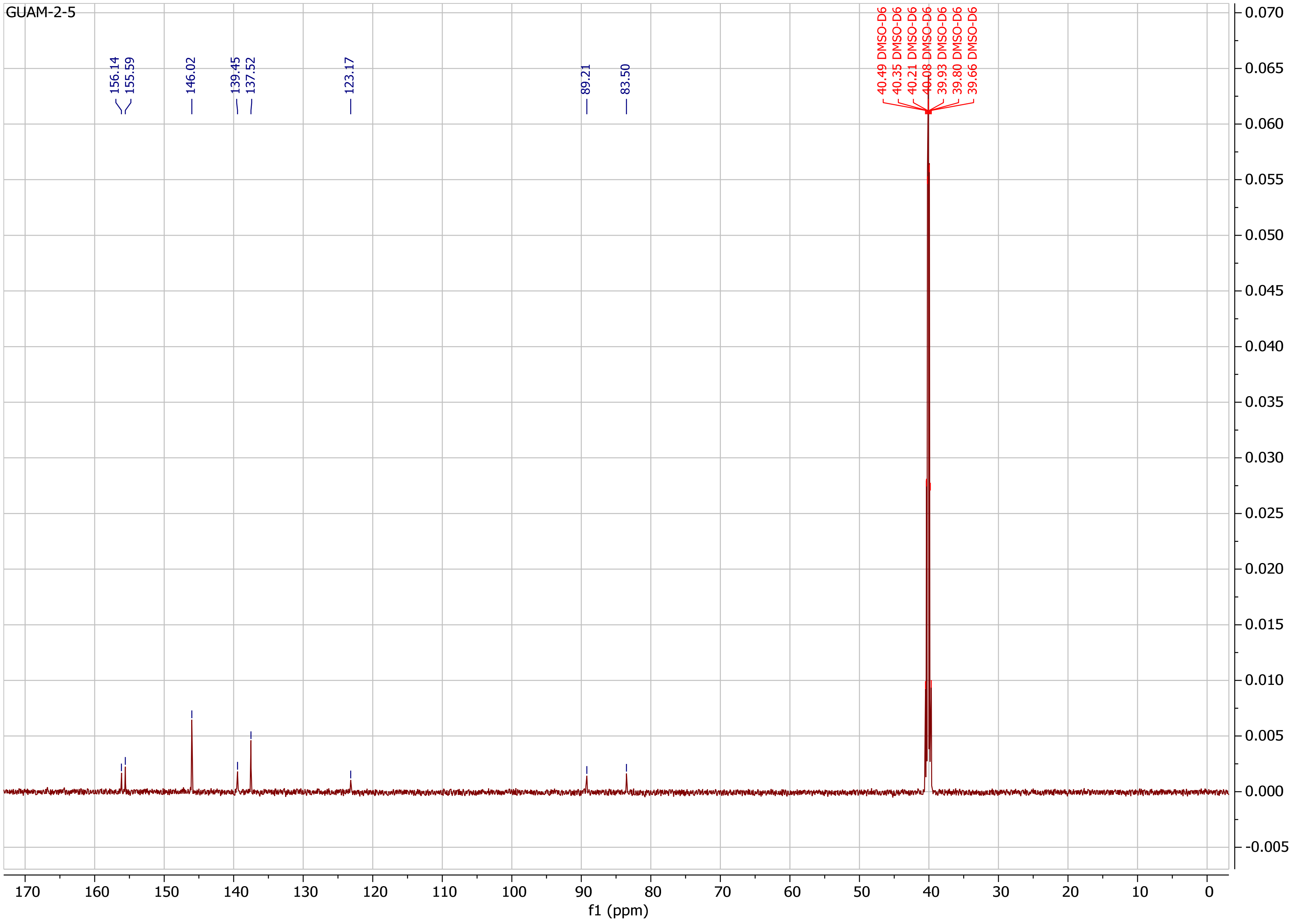

Supplement: Supplementary file 1 [file pharmaceuticals-14-01229-s001.zip › pharmaceuticals-1405634-supplementary.pdf]
